# Anticancer Activities of Marine-Derived Phenolic Compounds and Their Derivatives

**DOI:** 10.3390/molecules27041449

**Published:** 2022-02-21

**Authors:** Dario Matulja, Filip Vranješević, Maria Kolympadi Markovic, Sandra Kraljević Pavelić, Dean Marković

**Affiliations:** 1 Department of Biotechnology, University of Rijeka, Radmile Matejčić 2, 51000 Rijeka, Croatia; dario.matulja@biotech.uniri.hr (D.M.); filip.vranjesevic@biotech.uniri.hr (F.V.); maria.kolympadi@biotech.uniri.hr (M.K.M.); 2 Faculty of Health Studies, University of Rijeka, Viktora Cara Emina 5, 51000 Rijeka, Croatia

**Keywords:** marine natural products, phenolics, anticancer

## Abstract

Since the middle of the last century, marine organisms have been identified as producers of chemically and biologically diverse secondary metabolites which have exerted various biological activities including anticancer, anti-inflammatory, antioxidant, antimicrobial, antifouling and others. This review primarily focuses on the marine phenolic compounds and their derivatives with potent anticancer activity, isolated and/or modified in the last decade. Reports on the elucidation of their structures as well as biosynthetic studies and total synthesis are also covered. Presented phenolic compounds inhibited cancer cells proliferation or migration, at sub-micromolar or nanomolar concentrations (lamellarins D (**37**), M (**38**), K (**39**), aspergiolide B (**41**), fradimycin B (**62**), makulavamine J (**66**), mayamycin (**69**), N-acetyl-N-demethylmayamycin (**70**) or norhierridin B (**75**)). In addition, they exhibited anticancer properties by a diverse biological mechanism including induction of apoptosis or inhibition of cell migration and invasive potential. Finally, phlorotannins **1**–**7** and bromophenols **12**–**29** represent the most researched phenolic compounds, of which the former are recognized as protective agents against UVB or gamma radiation-induced skin damages. Finally, phenolic metabolites were assorted into six main classes: phlorotannins, bromophenols, flavonoids, coumarins, terpenophenolics, quinones and hydroquinones. The derivatives that could not be attributed to any of the above-mentioned classes were grouped in a separate class named miscellaneous compounds.

## 1. Introduction—Marine Natural Products as Anticancer Agents

The malignant diseases have represented one of the greatest challenges to the modern society and health care systems, and have induced the extensive research and clinical investigations by both the pharmaceutical industry and the scientific community [1]. According to Bray et al., around 18 million new cases were diagnosed worldwide in 2018 with more than a 50% mortality rate [2]. The initiation and progression of cancer are complex biological processes that involve several steps, leading to heterogeneity of tumor tissue and the microenvironment [3]. As reported by Hanahan and Weinberg, there are ten characteristic hallmarks of cancer which are required for its growth and spreading, including maintenance of proliferation alongside avoidance of growth suppressors, resistance to cell death, occurrence of replicative immortality, inducement and activation of angiogenesis, invasion and metastasis. Furthermore, cancer cells are characterized by genome instability and mutations being able to alter metabolism and promote inflammatory states while avoiding immune defense systems [4]. The most cancer-related deaths are caused by the migration of transformed cells from the site of the primary tumor to the distinct part of the organism which contributes to the reduction of the effectiveness of cancer treatment [5]. As discussed by Fife and colleagues, the metastatic cascade process results in the reorganization of the cytoskeleton, making actin and tubulin an important target for novel chemotherapeutic agents [6]. Besides metastases, targeting a hypoxic tumor environment and cancer stem cells are new promising bases for therapy improvement, as summarized by Lefranc et al. [3] In addition, the authors highlighted the need for the isolation and/or synthesis of new compounds that destroy cancer cells via a non-apoptotic effect since many types of cancer successfully avoid apoptosis [4]. Nevertheless, standard cancer treatment still involves the employment of cytotoxic or more selective anticancer compounds along with surgical procedures and radiation [7].

Notwithstanding the fact that a great progress has been made in the treatment of malignancies arising from the development of combination therapy and immunotherapy as a form of personalized medicine, traditional chemotherapeutics are still the most widely used. However, due to insufficient selectivity towards cancer cells, together with the developed resistance, they continue to cause severe side effects, weakening the immune system of the organism [8,9,10]. Consequently, the search for new bioresources of natural products with high anticancer activities have been further continued. As pointed out by Rocha and co-workers, almost 50% of current antitumor pharmaceuticals have been derived from natural products and their structures have served as starting points in the optimizations by SAR studies [7]. Unlike terrestrial, marine species are far less explored as the majority of the research began with the development of submarine technology from the mid-20th century and isolation of the first antitumor compound, spongothymidine [11]. The National Cancer Institute has evaluated that only 0.01% of metabolites isolated from terrestrial sources exhibited cytotoxic effects against cancer cells that can be compared to approximately 1% from the aquatic systems [12]. The latter can be explained by the presence of biological and chemical diversity of marine flora and fauna as a result of special and hard sea conditions, as well as external factors (pH, sunlight, temperature, salinity, predatory species) [11,13,14]. Consequently, marine micro- and macroorganisms produce secondary metabolites of unique and complex chemical structures, particularly recognizable by the high degree of oxygenation, unsaturation and/or the presence of halogen atoms, which can be rarely found in terrestrial natural products [15,16]. Many reviews have outlined a wide range of biological activities displayed by marine natural products, including antioxidant, antimicrobial, anti-inflammatory and anticancer, and their potency as new candidates of pharmaceutical, nutraceutical and cosmeceutical interest [14,16,17]. Finally, in the last decade, it has been shown that bacteria and cyanobacteria, living in a symbiotic relationship with sedentary macroorganisms, could also modify or produce their own bioactive metabolites, from which are few already approved or in a clinical trial [18].

Up to date, there have been approved 12 drugs for cancer treatment, mostly as synthetic analogues of marine natural products (alkaloids, peptides, macrolide polyketide, nucleosides and indolocarbazole): cytarabine (Cytosar-U^®^, 1969), nelarabine (Arranon^®^, 2005), trabectedin (Yondelis^®^, 2007), fludarabine phosphate (Fludara^®^, 2008), eribulin mesylate (Halaven^®^, 2010), brentuximab vedotin (Adcetris^®^, 2011), midostaurin (Rydapt^®^, 2017), plitidepsin (Aplydin^®^, 2018), polatuzumab vedotin (Polivy^®^, 2019) and enfortumab vedotin (Padcev^®^, 2019) [19,20,21,22]. Additionally, in 2020, lurbinectedin (Zepzelca^®^) and belantamab mafodotin (Blenrep^®^) were approved by FDA (U.S. Food and Drug Administration) as alkaloid and monoclonal antibody-peptide conjugate, respectively [23,24]. As mentioned by Jimenez and her group, dozens of new candidates connected to marine natural products undergo all phases of clinical trials, of which drug-antibody conjugates are the most represented [25]. The number of medicines on the market is likely to grow in the coming years. In this review, phenolic compounds of marine origin isolated or studied as anticancer agents in the last decade are presented. Their natural resource and metabolomic origins, isolation methodologies, synthesis alongside with in vitro and in vivo anticancer activities are also summarized.

## 2. Phenolic Compounds of Marine Origin—General Characteristics and Biosynthesis

Phenolic metabolites are organic compounds with at least one or more hydroxyl groups attached to arylic systems with simple variations to highly polymerized molecules [26,27]. They are often found in terrestrial plants as well as in red, brown and green macroalgae (seaweeds) [26], sponges [28,29], corals [30,31,32,33] and microalgae [34]. The recent studies have shown that marine-derived microorganisms also produce these phytochemicals [35], what could be confirmed by a metagenomic analyses [36]. In addition to their primary role in plant physiology for structural scaffolding and maintenance of the plant integrity as well as in the development of the plant through lignin and pigment biosynthesis, the phenolic compounds have an important role as secondary metabolites in protection against stress, defence against predators, repellent or extermination of microorganisms or other pathogens, the fouling process and UV radiation [26]. Phenols’ structural versatility is the result of modifications of the basic phenolic structure obtained through glycosylation, oxygenation, hydroxylation, methylation, unsaturation, isomerization, and oligomerization or polymerization [37], as well as the variations in the oxidation state of the pyran ring as observed for flavonoids [38]. Many of the natural phenols are glycosylated while the toxic effect is exerted by the aglycone part [39]. Due to unique structures and diverse scaffolds containing halogen atoms and the possibility of polymerization into multiple forms, all contributing to broad pharmacological activities, phenolic compounds of marine origin have attracted the great attention of the scientific community [26].

The most recent and comprehensive review by Mateos and colleagues presented an insight into marine phenolics regarding their antidiabetic, antioxidant, antiviral, antimicrobial, anti-inflammatory and anticancer activities [40]. However, their activities are largely affected by pharmacokinetic and pharmacodynamic properties, especially bioavailability, absorption and metabolic enzymatic interactions. Some of those are observed only in in vivo studies, since in vitro assays might result in inadequate results and conclusions [38]. Poor bioavailability of phenols is mostly due to rapid and extensive metabolism and rather poor passage through the blood brain barrier or, in case of quercetin, the low absorption of the bioactive aglycone part in the gastrointestinal tract [41]. Moreover, certain phenolic acids like caffeic or syringic acid, as well as coumarin derivatives, were predicted by in silico methods to be more hydrophobic, thus capable to pass the brain barrier to a greater extent [42].

Regardless of the therapeutic potential of this class of compounds, it should be noted that phenolics from marine species are significantly less explored than their terrestrial counterparts. Their isolation is often challenging because of the instability and the high chemical reactivity, and often, the additional necessity for the employment of new technologies for the isolation as well as the establishment of an appropriate analytical identification and characterization method [27]. In a recent review, Getachew and co-workers compared novel and green extraction processes with the conventional techniques that are usually time consuming and use the large amounts of organic solvents. The authors showed that the employment of the extraction procedures assisted by ultrasound, microwave or enzyme as well as pressurized liquid and supercritical fluid (ScCO_2_) extractions gave phenolics in higher isolated yields in a shorter time and with a smaller consumption of solvents compared to the classical extraction methods [43].

Organism collection and supply, together with low yields and small amounts of isolated natural products, are the main challenges in marine bioprospecting. The development of the efficient syntheses of the desired biologically active metabolites, with the aim of preserving the marine ecosystem, is thus often essential [44]. The understanding of secondary metabolites biosynthesis in organisms could aid to the establishment of bioinspired synthesis and development of new synthetic strategies [45].

**Scheme 1:**
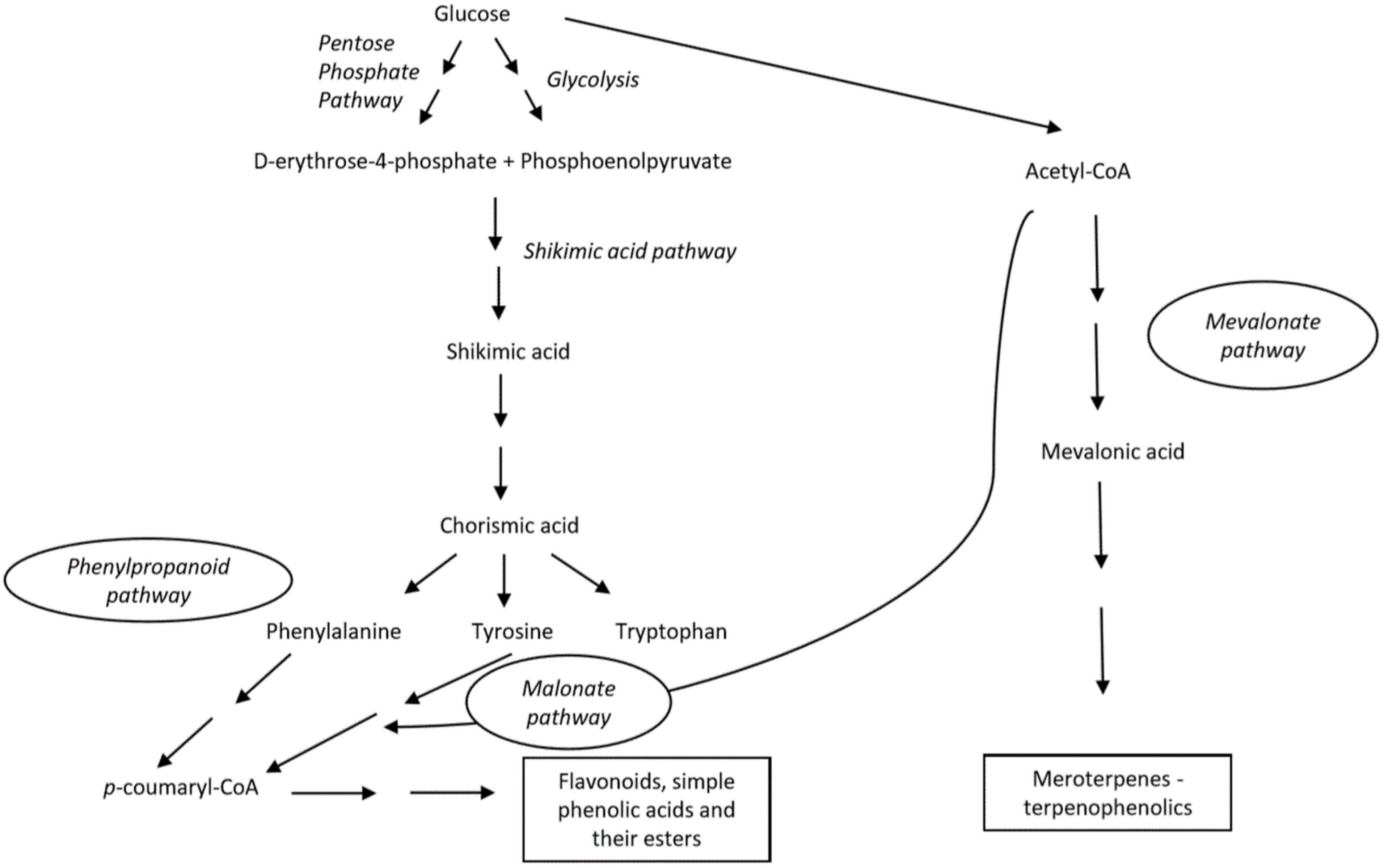
Main biosynthetic pathways for generation of phenolic compounds.

Phenols in nature can be synthesized through three known pathways, connecting the metabolism of primary carbohydrates and amino acids with secondary metabolites: the shikimic acid-phenylpropanoid pathway, the malonate-acetate (polyketide) pathway and mevalonate-acetate (isoprenoid) pathway (Scheme 1) [46,47]. The malonic acid pathway is significantly expressed in microorganisms, both, fungi and bacteria [48]. The shikimic acid-phenylpropanoid pathway is the major biosynthetic route for the formation of most of phenols, especially in vascular plants.

The shikimate pathway initially involves the synthesis of aromatic amino acids (phenylalanine, tyrosine and tryptophan) via the modification of chorismic acid, obtained from carbohydrate precursors in glycolysis. The biosynthesis of phenylpropanoid partly involves distinct enzymes that might be differently expressed depending on both, biotic and abiotic features, and thereby can induce additional complexity to the produced compounds. The key enzyme is phenylalanine ammonia-lyase (PAL), which is responsible for the formation of cinnamic acid. Next, cinnamic acid is transformed into *p*-coumarid acid by cinnamate 4-hydroxlyase (C4H) [45,48,49].

Similarly to previous pathways, in the malonic acid pathway, the tyrosine is deaminated to *p*-coumaric acid by the key enzyme, tyrosine ammonia lyase (TAL). In addition, *p*-coumaric acid is functionalized by CoA to produce *p*-coumaroyl-CoA by 4-coumaroyl CoA ligase (4CL) and further reacts with three molecules of malonyl-CoA obtained from acetyl-CoA to give chalcone by chalcone synthase. The obtained tetraoxychalcone is transformed to flavanone–naringenin that serves as a precursor of many other flavonoids [50,51]. By both of these pathways, flavonoids, simple phenolic acids and their esters are being produced.

Kumari et al. summarized the mevalonate-acetate isporenoid pathway in which basic isoprene (C_5_) units are formed [52]. The latter can be also obtained via a non-mevalonate route. Further condensation of such units in linear or branched intermediates that, eventually, undergo multi-step cyclization reactions catalyzed by terpene synthases and generate miscellaneous terpenoids. As authors elaborated, such a biosynthetic pathway leads to the formation of isoprene units as fundamental structural elements of terpenoids, but also phenols and alcohols [52].

The biosynthesis of natural phenolics in terrestrial plants has been more studied and better understood compared to biogenesis of phenols present in marine organisms [27]. In recent decades a significant progress has been made regarding that issue [53,54,55]. For example, Mandrekar and colleagues have reviewed the mechanism of formation of brominated metabolites, including bromophenols (Section 3.2). The role of vanadium haloperoxidases that are responsible for the incorporation of bromine in marine natural products was highlighted. The reaction occurs in two steps, including the generation of enzymatically formed BrOH which then promotes an electrophilic attack (bromination) of certain organic molecules [53]. Another interesting group of marine phenolic compounds, phlorotannins (Section 3.1) are formed by the polymerization of 1,3,5-trihydroxybenezene units (phloroglucinol, **1**) into more complex structures [54]. However, the complete mechanism is poorly understood and remains to be discovered, despite Meslet-Cladière et al. proposing the key step in phlorotannin biosynthesis by analyzing the brown alga, *Ectocarpus siliculosus*. As demonstrated by the authors, a polyketide synthase (type III, PKS) is essential for the synthesis of the phloroglucinol unit from malonyl-CoA of the malonic acid pathway. Further, they observed a positive correlation between the PKS gene and the quantity of the phloroglucinol compound [55].

## 3. Anticancer Properties of Marine-Derived Phenolic Compounds

Marine-derived phenolics exhibit a wide range of biological effects as a consequence of structural uniqueness and complexity [40]. According to Jimenez-Lopez et al., those effects arise mostly from their antioxidant activity, in particular, the ability to scavenge reactive oxygen species (ROS) by a direct H-atom donation process or single electron transfer (SET) from the hydroxyl groups followed by protonation [56]. The latter has been extensively computationally studied regarding the thermodynamic and kinetic properties of the transfer reaction by Di Meo and co-workers [57]. Furthermore, since redox homeostasis is impaired in tumor tissue, which at a certain (moderate) concentration can initiate the growth and progression of cancer cells, ROS represents a potential target for chemotherapeutics [58]. On the other side, anticancer activity of marine phenols could be achieved by the suppression of the telomerase expression and activity or by the modulation of the proliferation signal pathways as reported and reviewed by others [40,59]. Numerous research studies have been conducted so far, investigating the anticancer activity of organic or water extracts obtained mostly from macro- and microalgae, sponges and tunicates [60,61,62,63,64,65,66,67,68]. As admonished by Rosa et al., it is necessary to characterize the chemical composition so that the activity can be associated with the presence of a certain metabolite. The same authors have pointed out a scarce number of in vivo studies and clinical trials of phenolic metabolites to date [69].

As the backbone in the development of new drugs, this review aims to provide an overview of the recently isolated and characterized marine phenolic compounds together with their most significant antitumor effects. Phenolic metabolites are divided into seven classes as following: phlorotannins, bromophenols, flavonoids, coumarins, terpenophenolics, quinones and hydroquinones and miscellaneous compounds. Importantly, the nomenclature of compounds, more precisely the numbering of the positions of atoms and substituents, is retained as in the original research papers. Some of the compounds can be classified into several different groups, confirming a complex division of natural-derived products.

### 3.1. Phlorotannins

**Figure 1:**
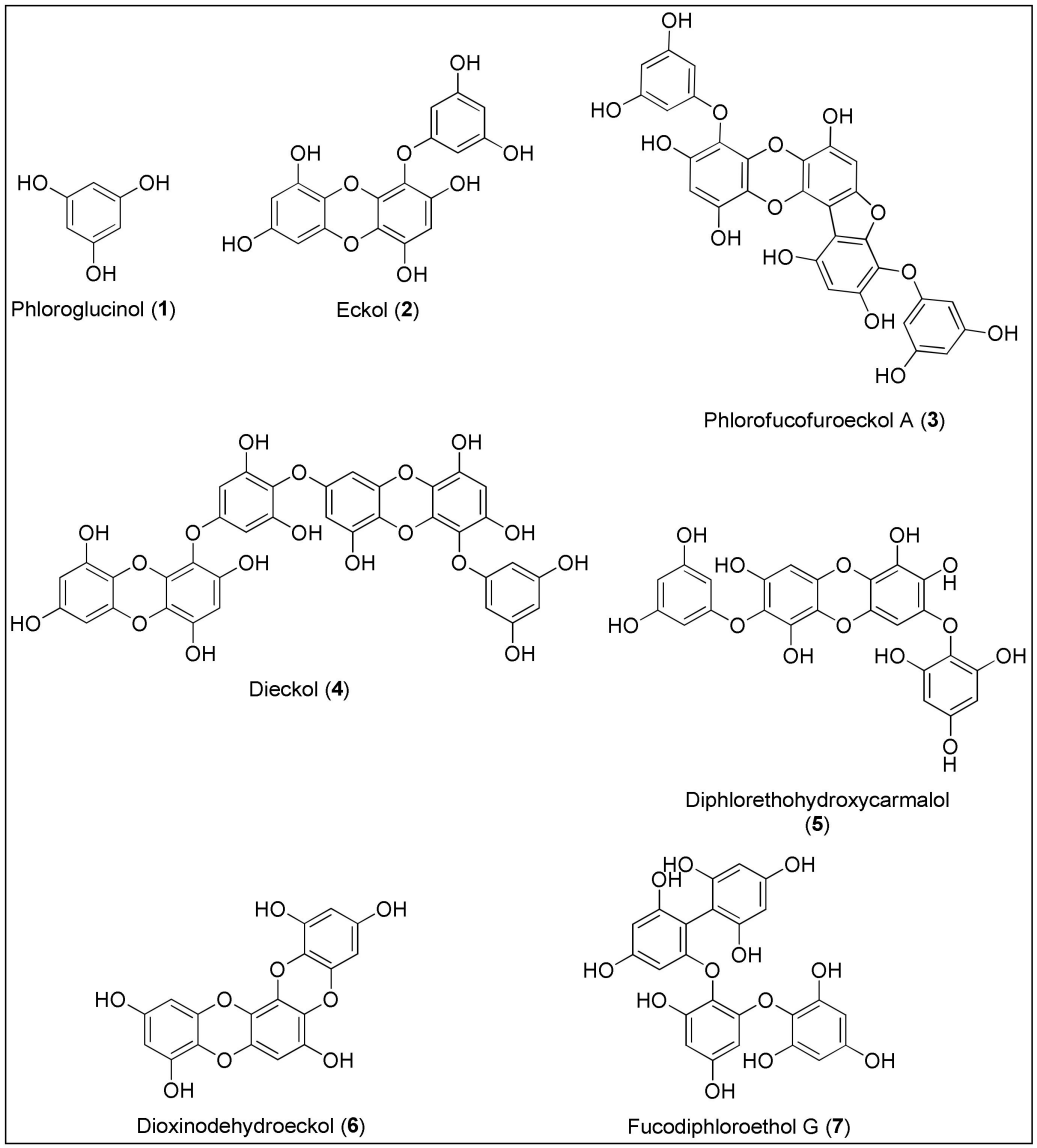
Chemical structures of phlorotannins **1**–**7** with anticancer activities isolated from brown seaweeds.

Phlorotannins **1**–**7** are a class of phenolic compounds similar to terrestrial-derived tannins, recognizable by the high proportion of hydroxyl groups with elevated water solubility and a binding affinity for the biological macromolecules such as proteins and polysaccharides (Figure 1) [69,70,71,72]. They are biosynthesized by marine brown macroalgae (Phaeophyta) and can be considered as primary and secondary metabolites, with the former resulting from their role in the formation of the cell wall based on the phlorotannin-alginic acid complex. Their secondary metabolite functions include protection from infections and ultraviolet radiation by high absorbance of the UV-B spectrum, as well as defense against herbivores [70]. Phlorotannins are formed by the polymerization ofna monomer unit, phloroglucinol (**1**), thereby achieving a wider mass range when compared to the terrestrial counterparts. The monomers can combine through carbon-oxygen or carbon-carbon bonds at different positions and to different degrees, forming linear, branched and cyclic compounds [70]. Such structures of phlorotannins are highly susceptible to pH, light, temperature and oxidation, causing an urgent need to develop new technologies in the field of nano science and green chemistry strategies [73]. Therefore, a novel method of encapsulation has been investigated using polyvinylpyrrolidone nanoparticeles, which resulted in enhanced functions of phlorotannins with regards to a reduced kinetic release and toxicity towards normal cells and greater antiradical activity [74]. Just recently, Kaushalya and Gunathilake have isolated phlorotannins from the brown algae, *Sargassum ilicifolium*, and performed encapsulation in the chitosan-tripolyphospahte carrier. Those particles were found to exhibit higher antioxidant activities and total phlorotannin content upon in vitro digestion, indicating the potential for the targeted delivery of phlorotannins [75].

Phlorotannins are classified into four main groups based on the linkage functionality between aromatic units. Fuhalols and phlorethols possess an ethereal linkage, fucols aromatic, fucophlorethols ethereal and aromatic linkages, whereas eckols and carmalols are bridged through a 1,4-dioxin linkage [72]. Due to biological potential and the possibility of the atropoisomeric chirality because of the restricted rotation, these molecules have attracted considerable attention of synthetic chemists [76]. As stated by Erpel and her team in a comprehensive review on phlorotannins’ isolation and therapeutic potential, this class of phenolics exerts anticancer activity by various biological mechanisms, leading to apoptosis and inhibition of angiogenesis and tumor metastasis [71].

Phloroglucinol (**1**) was primarily isolated from marine brown alga, *Ecklonia cava* (Kjellman, 1885), but it is also produced by other species belonging to the classes of Phaeophyceae and Fucaceae [77,78]. Phloroglucinol (**1**), displayed antioxidant, antibacterial, enzyme inhibition and anticancer activities [70]. In 2012, Kwon and colleagues were the first who conducted a study on phloroglucinol’s potential to inhibit tumor angiogenesis in vitro and in vivo. As reported, phenol **1** inhibited the migration and capillary-like tube formation of endothelial progenitor cells’ processes depending on the vascular endothelial growth factor (VEGF) which resulted in the reduction of Lewis lung carcinoma in a mouse model. To obtain more information regarding cancer growth and metastatic spread, the additional evaluation of the mechanism that triggers the inhibition has to be conducted [79]. Although phenol **1** displayed 50% inhibition on the invasive and migratory ability of the MDA-MB-231 breast cancer cells at a concentration of 50 µM, Kim et al. reported that it did not initiate their significant death. The authors also demonstrated that treatment with **1** led to the downregulation of SLUG protein in the aforementioned MDA-MB-231 and BT549 breast cancer cell lines, via PI3K⁄Akt and Ras⁄Raf-1⁄ERK signalling pathways. As suggested, SLUG is responsible for the regulation of epithelial-mesenchymal cell transition, and thus for invasiveness of cancer cells [80]. The Kang’s group conducted two studies in 2014, invetigating the pro-apoptotic activity of phenol **1** against HT-29 colon cancer cells [81,82]. They observed that apoptosis of the cells occurred through an insulin-like growth factor 1 receptor (IGF-1R) and the inhibition of downstream proteins, similarly to the results of Kim et al. [82]. Phenol **1** also altered the expression of proteins belonging to the Bcl-2 family; more precisely, it induced the upregulation of pro-the apoptotic proteins Bax and Bad and the downregulation of anti-apoptotic Bcl-2 and Bcl-xL [81]. In 2015, Kim and co-workers reported that **1** exhibited anticancer activity against breast cancer stem-like cells by reducing the expression of CD44, Oct4, Notch2, β-catenin and Sox2 proteins. The latter is often connected to the development of resistance against chemotherapeutics. The treatment with phenol **1** enhanced the sensitivity of cells to the ionizing radiation and standard chemotherapeutic drugs (taxol, cisplatin and etoposide) [83]. The higher sensitization of colon cancer cells, HT-29 and HCT116, to 5-fluorouracil (5-FU) was also observed by Lopes-Costa et al. after treatment with **1**. Interestingly, the authors also noticed that phloroglucinol (**1**) decreased apoptosis of cells at a lower concentration which can be explained by the scavenging activity of phenol against ROS, induced by 5-FU [84].

Another phlorotannin isolated from *Ecklonia cava*, eckol (**2**), was found to exhibit radioprotective properties in vitro by ROS scavenging [85] and in vivo through the reduction of side effects caused by gamma ray-irradiation of entire mouse bodies, more precisely, the repair of damaged DNA and the recovery of hematopoiesis [86]. Based on these results, Park’s group suggested that eckol (**2**) could be used as an adjuvant therapy to mitigate the side effects of radiation in cancer patients [85,86]. In 2011, Hyun et al. assayed **2** for targeting stem-like glioma cells. Eckol (**2**) decreased the expression of CD133, Nestin and Musashi-1, which represent markers of stem cells. Further, treatment with **2** increased the sensitivity of glioma cells to the cytotoxic prodrug, temozolomide, as well as to ionizing radiation via PI3K⁄Akt and Ras⁄Raf-1⁄ERK signalling pathways, similarly to phloroglucinol (**1**) (vide supra) [87]. Moreover, phlorotannin **2** significantly inhibited the growth of the pancreatic cancer cell line (SW1990) induced by the presence of an exogenous regenerating gene protein (Reg3A). The biological importance of the Reg3A protein is evident in the influence on pancreatic cancer cells’ proliferation and progression, additionally with the appearance of an inflammatory microenvironment [88]. Furthermore, another member of pancreatitis-associated proteins, Reg3g, was upregulated in an experimental colitis mouse model when treated with eckol (**2**) at doses of 0.5–1.0 mg/kg. Since Reg3g appeared to be a protection against the colon injury development, the authors suggested the possibility of using **2** as an alternative treatment of colitis [89]. A relation between the pharmacological effects of eckol (**2**) and the modulation of the innate and adaptive immune systems was also observed by Zhang and co-workers in vivo on a mouse model bearing a sarcoma (S180) xenograft. Namely, a phlorotannin **2** administration resulted in the increase of cytotoxic T-lymphocytes, the induction of a type 1 helper T cell (Th1), which is required for the annihilation of the tumor and the activation of dendritic and mononuclear phagocytic cells [90]. Finally, Cho et al. very recently demonstrated protective activity of eckol (**2**), in vitro, against skin inflammation promoted by a mixture of TNF-α and IFN-γ, further emphasizing the importance of **2** in inflammatory processes and responses [91].

In 2012, Eo et al. performed a mechanistic study regarding the induction of apoptosis in human colorectal cancer cell lines with the derivative of eckol (**2**), phlorofucofuroeckol A (**3**) [92]. The results showed that at 100 μM, there were **3** decreased cells viabilities of 38%, 31%, 47% and 90%, for HCT116, HT-29, LoVo and SW480 cancer cells, respectively, with the SW480 cells being the most sensitive to **3**. Furthermore, the authors assumed that cell viability might have occurred via apoptosis since its biomarker, cleaved poly (ADP-ribose) polymerase (PARP) was increased. Furthermore, treatment with **3** led to the enhanced expression of a possible therapeutic target, activating transcription factor 3 (ATF3) [92]. Lee’s group extracted phlorofucofuroeckol A (**3**) from *E. cava* and observed the suppression of migration and invasion of MCF-7 and MDA-MB-231 breast cancer cell lines by the downregulation of Nf-κB activity and Toll-like receptor 4 (TLR-4) signalling. The latter is shown to be crucial in the development of breast cancer. Moreover, the results indicated that **3** reduced the expression of matrix metalloproteinases (MMPs) 2 and 9, also involved in invasion and metastasis processes [93]. The most recent study carried out by Manandhar et al., investigated the activity of **3**, isolated from *Ecklonia stolonifera*, against a mouse melanoma cell line B16F10. However, phlorofucofuroeckol A (**3**) did not exhibit a significant antiproliferative effect towards the above mentioned cancer cells [94].

Between all phlorotannins, the anticancer properties of dieckol (**4**) have been the most comprehensively evaluated. Similarly to phlorofucofuroeckol A (**3**), dieckol (**4**) also induced a reduction in invasive potential of MCF-7 cells [93]. Zhang and colleagues studied the cytotoxicity of **4**, the effect on invasiveness of human fibrosarcoma cells (HT1080) as well as its influence on the expression of MMP-2 and -9. The treatment of HT1080 cells with dieckol (**4**) did not result in a general toxic outcome even at the highest concentration of 250 µM. Furthermore, **4** also downregulated the expression of the tested matrix metalloproteinases through Nf-κB signalling [95]. Interestingly, analogous observations were made by Oh and co-workers during the examination of the inhibitory effect of dieckol (**4**) against hepatocellular carcinoma cells (SK-Hep1). The authors also reported that the negative effect of **4** on MMP-9 could have arisen from the modulation of the MAPK signalling pathways [96]. Importantly, very recently all molecular mechanisms underlying MMP inhibition (MAPK, activator protein-1 and NF-κB signalling) were confirmed by Wang’s group by using a human dermal fibroblast cell line (HDF) [97]. In 2012, Park et al. performed two studies and analyzed a new anticancer strategy by employing dieckol (**4**), regarding the inhibition of ROS and migration potential of the malignant and non-metastatic melanoma cells, B16F10 and B16F0, respectively [98,99]. The authors observed that the higher concentration of ROS (in particular, H_2_O_2_) enhanced the migration of B16F0, which was in relation to increased levels of the Ras-related C3 botulinum toxin substrate 1 (Rac1) and the Wiskott–Aldrich syndrome protein family member 2 (WAVE2). Contrary, treatment with **4** diminished those effects accompanied by the inhibition of actin polymerization [98]. Both proteins are included in the signalling pathway associated with actin reorganization and cell motility and, consequently, the metastasis of cancer cells [100]. The latter is in agreement with the results from the second study where **4** suppressed the migration of HT1080 cells via reduced expression and phosphorylation of focal adhesion kinase (FAK) which is also related to the migration potential of the cell [99]. Dieckol (**4**) can also cause the selective anticancer activity against hepatocellular carcinoma cell lines (Hep3B, Sk-Hep1). At a concentration of 100 µM, **4** induced apoptosis of 62.2% of Hep3B cells. Furthermore, Yoon et al. proposed signalling pathways, underlying apoptosis, which are manifested in the activation of mitochondrial-related release of cytochrome *c* and death receptor-mediated caspase cascade as revealed by immunoblotting analyses [101]. In 2015, an interesting study was conducted by Li and co-workers in which dieckol (**4**) acted as antiproliferative and antiangiogenic agent through the inhibition of the mentioned MMP-2 and -9, MAPK signalling proteins, ERK, and p38, additionally targeting VEGF. The authors used non-transformed, human umbilical vein endothelial cells (EA.hy926), of which the viability was not significantly decreased by **4**. In vitro results were compared to computational docking calculations which showed that **4** accomplishes binding to aforementioned proteins via predominantly formed hydrogen bonds with certain amino acids [102].

The antitumor activity of the dieckol (**4**), resulting from the influence of this phlorotannin on proteins involved in the apoptotic signalling pathway and from the induced oxidative stress, was also studied by Ahn’s team [103]. They reported the IC_50_ values of 77.3 and 92.7 µM in ovarian cancer cells, A2780 and SKOV3, respectively. Therewithal, the antitumor efficacy was also evaluated in vivo using mice bearing ovarian tumors, the volume of which was significantly decreased after a four-week treatment with **4**, without inducing side effects in terms of liver and kidney toxicity [103]. Moreover, in 2015, Kim’s group applied the centrifugal partition chromatography to isolate three members of eckol subclass of phlorotannins from *E. cava*. Among them, dieckol (**4**) exhibited the highest anticancer activity against human breast cancer cell line, MCF-7 cells, wherein the IC_50_ value was between the two highest concentrations (64 and 128 µM). The gap-closure assay demonstrated that the mobility of the cells was significantly lower upon treatment with **4**. That observation was corroborated by the increased expression of glycoproteins, TIMP-1 and -2 which are inhibitors of MMP and involved in the degradation of extracellular matrix (ECM), thus contributing to the suppression of cell migration. These results also matched with the downregulation of MMP-9 [104].

You et al. used the cell proliferation reagent (WST-1) to examine the dependence of viability of MCF-7 and SK-BR-3 breast cancer cells on various concentrations of dieckol (**4**). Contrary to the Kim’s report from 2015 mentioned before, 50% of cell death was reached at concentration values higher than 200 µM. An increase in Bax/Bcl-2 ratio indicating mitochondrial-mediated apoptosis was observed, but only in SK-BR-3 cells. Thus, further studies have to be undertaken [105]. Phlorotannin **4** was found to exert protective and chemopreventing effects against hepatocellular carcinoma (HCC) in rats induced by *N*-nitrosodiethylamine (NDEA) administration, as reported by Sadeeshkumar et al. [106,107]. At a dosage of 40 mg/kg, **4** prevented lipid peroxidation, increased antioxidant activity and reversed the activities of certain hepatic marker enzymes which the authors hypothesized as a consequence of scavenging capacity of dieckol (**4**) [107]. The same group analyzed the molecular mechanisms that mediated observed NDEA-induced hepatocarcinogenesis in male Wistar rats. Phlorotannin **4** also displayed potential chemoprevention through the modulation of angiogenesis, invasion, apoptosis and inflammation via the upregulation of VEGF, MMP-2/9, proliferating cell nuclear antigen (PCNA) and COX-2 (cyclooxigenase-2), respectively [106]. In addition to breast, melanoma and hepatocellular cancer cells, dieckol (**4**) was also screened for anticancer properties against non-small-cell lung cancer (A549) [108] and human pancreatic cancer cells (PANC-1) [109] in the two most recent studies. At concentrations of 25 µg/mL and 20 µM, **4** induced 50% of A549 and PANC-1 cells deaths, respectively [108,109]. Wang and colleagues demonstrated the anti-migratory and apoptotic activity of **4** in A549 cells, associated with the inhibition of the Pi3K/AKT/mTOR signalling pathway and activation of the tumor-suppressor, E-cadherin [108]. Lastly, Xu and co-workers proved that **4** could be used to treat pancreatic cancer since it increased the expression of proapoptotic protein (Bax) and decreased antiapoptotic Bcl2 protein as well as cyclin D1, whose overexpression is often related to chemoresistance [109]. Finally, deickol (**4**) was found to provide the protection from gamma radiation and consequent damage, both in vitro and in vivo [110,111].

In 2004, Toume et al. isolated and characterized diphlorethohydroxycarmalol (**5**) from another brown alga, *Ishige okamurai*. Carmalol derivative **5** displayed moderate cytotoxic activity towards vincristine-resistant as well as sensitive murine leukemia cells (P-388) with reported IC_50_ values being 8.0 and 10.5 µg/mL [112]. Yeon’s group was the first to assess the anticancer activity of **5** against promyelocytic leukemia cells (HL60) and study its apoptotic mechanism. According to the authors, the compound **5** induced concentration-dependent growth inhibition with the IC_50_ value lower than 25 µg/mL. Western blot analysis demonstrated that the treatment of HL60 cells with **5** resulted in an increased expression of cleaved caspase-3 and cleaved PARP as biomarkers for cell apoptosis. Furthermore, the upregulation of pro-apoptotic Bax and downregulation of anti-apoptotic Bcl-2 were also observed [113]. Similarly, these results were also confirmed by Park et al. by using the mouse embryonic fibroblast cell line (3T3-L1) [114]. Interestingly, several studies indicated photoprotective activity of diphlorethohydroxycarmalol (**5**) against UVB radiation and side effects, through the modulation of antioxidant system, absorption of radiation, inhibition of MMPs expression and scavenging of ROS, thus, demonstrating a wider possibility of biological action of **5** [115,116,117,118].

Another component of the *Ecklonia* species, an eckol derivative dioxinodehydroeckol (**6**), exhibited significant antiproliferative activity against MCF-7 with regard to MDA-MB-231 cells with 50% inhibition of proliferation induced at 5–10 and 50–100 µM, respectively. As studied by Kong et al., **6** induced apoptosis in MCF-7 cells via the regulation of the NF-κB pathway which eventually caused the downregulation of p65, IKK (IκB kinase) and NIK (NF-κB inducing kinase). The apoptotic activity of **6** was also associated with increased activities of caspase-3 and -9 and enhanced cleavage of a caspase substrate, PARP. The treatment with **6** induced a higher expression of pro-apoptotic Bax and lower expression of anti-apoptotic protein, Bcl-2 [119]. Contrary to these results and at the same time, Yoon and co-workers observed that the dioxinodehydroeckol (**6**) was neither effective on melanoma cells (B16F10) nor it affected melanin biosynthesis in melanoma cells [120]. Interestingly, Lee and colleagues came to opposite conclusions when screening for anti-melanogenic activity of **6** in B16F10 cells. According to the authors, the phlorotannin **6** inhibited melanin production and tyrosinase activity with reported IC_50_ values of 19.3 ± 1.2 and 24.2 ± 0.9 µM, respectively. Western blot and RT-PCR analyses revealed that the compound **6** downregulated tyrosinase and tyrosinase-linked proteins (TRP-1 and TRP-2) as well as MITF (microphthalmia-associated transcription factor), which are all involved in the regulation of melanin formation. Nevertheless, the viability of melanoma cells was not significantly changed by **6** [121]. The same modulation of apoptotic proteins and of the Bcl-2 family together with a caspase-dependent pathway was demonstrated by Ryu et al. in human keratinocyte cells (HaCaT) which were exposed to UV-B radiation. Based on those results, dioxinodehydroeckol (**6**) could be considered as a protective compound against radiation-induced skin damage [122].

Ham and colleagues were the first to isolate a phlorotannin metabolite with phenyl and ether bridges connecting phloroglucinol units, named fucodiphloroethol G (**7**), from a methanol extract of *E. cava* [123]. A comprehensive screening of antiproliferative properties of **7** against human cell lines was performed by Li’s group who noticed a specific activity towards cancer cells rather than a normal fibroblast cell line (MRC-5). At concentration values of 298.2, 226.5, 242.5 and 228.5 µM, fucodiphloroethol G (**7**) caused 50% inhibition of HeLa (human cervical cancer), A549, HT1080 and HT29 (colon carcinoma) cancer cells, respectively [124]. This was preceded by another research performed by Li et al., when assaying **7** against leukemia cells (HL-60). However, they used a much lower concentration (≤100 µM) and concluded that treatment with polyphenol **7** did not cause any significant cytotoxic effects against the aforementioned cell line [125]. Mostly the same research group conducted an extensive study of the molecular basis of angiogenesis suppression induced by **7** in human umbilical vein endothelial cells (EA.hy926 and ECV-304), in which angiogenic activity was stimulated by exogenous VEGF. Results have demonstrated that fucodiphloroethol G (**7**) blocked the MAPK and Akt signalling pathway necessary for the expression of proteinases (MMP-2, MMP-9 and APN) involved in the degradation of the extracellular matrix. Since the latter is crucial for invasion and cancer spreading, this signalling protein represents novel targets for therapies of metastatic cancers [126].

### 3.2. Bromophenols

**Figure 2:**
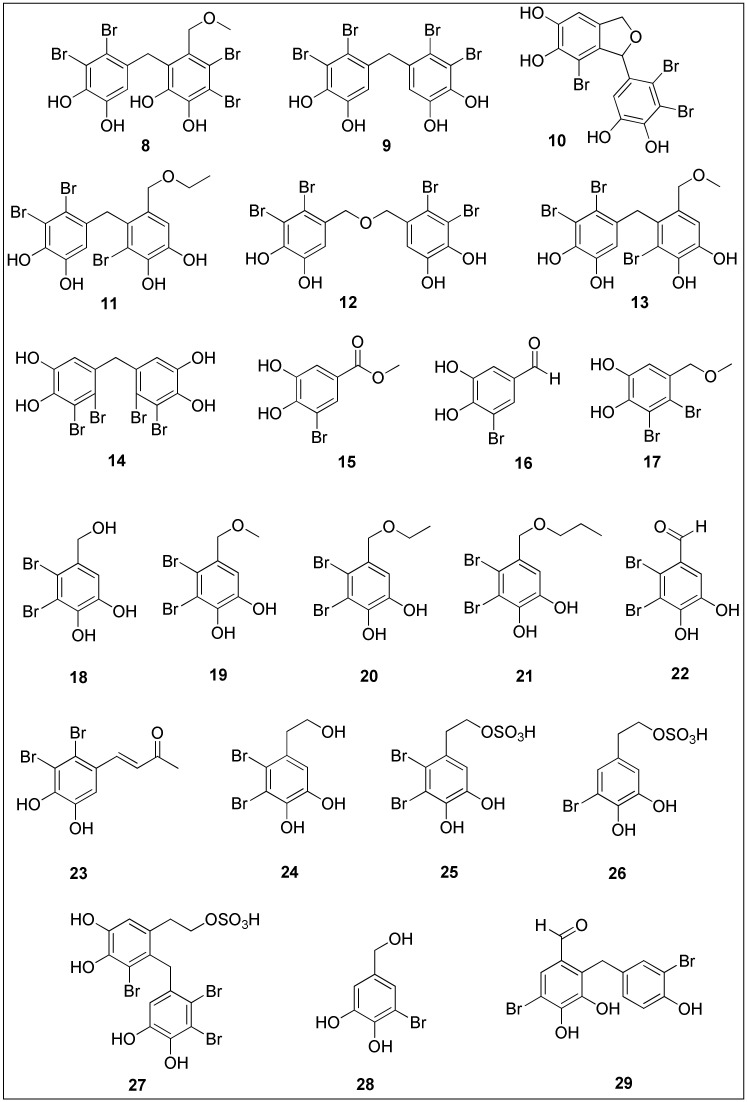
Chemical structures of bromophenols **8**–**29** with significant anticancer activities isolated from brown, red and green seaweeds.

More than 5000 halogenated natural products have been isolated and characterized, most of which are biosynthesized by various marine organisms (algae, corals, sponges and marine-associated microorganisms) [127]. The presence of halogen atom substituents often leads to better biological activities due to the possibility of the formation of various interactions between target and ligand, which include hydrogen and halogen bonds as well as other polar–polar interactions. The presence of halogen atoms may also cause conformational changes in molecules, the enhancement of lipophilicity and an increased affinity and binding constant of ligand to target, thus attracting synthetic and medicinal chemists to study and to develop functional foods as well as to design new drugs [128,129]. More than 600 halogenated compounds of a marine origin have been presented in a comprehensive review by Gordon W. Gribble, summarizing their antiparasitic, antiviral, anti-inflammatory, antibacterial, anticancer and antioxidant activities [127]. One of the interesting class of organohalogens are bromophenols **8**–**29** (Figure 2). These compounds can be characterized by the diverse phenolic skeletons bearing at least single bromine substituent [130,131]. Even though bromophenolics have been isolated from green, brown and red algae, the latter have been found to contain brominated compounds as one of the major class of metabolites [53].

Bromination can possibly lead to the improvement of bioselectivity [132]. For example, in 2004, Xu and co-workers [133] isolated several dibenzyl bromophenols **8**–**13** from an ethanolic extract of marine brown alga, *Leathesia nana*. These bromophenols exhibited moderate antiproliferative activity against several cancer cell lines with IC_50_ values in a nanomolar range, notwithstanding the fact that no activity of preliminary screened ethanolic extract was observed. At concentrations of 1.8, 3.8, 2.7 and 2.2 nM, bromophenol **9** inhibited 50% of A549, BGC-823, MCF-7 and HCT-8, respectively. Moreover, treatment of hepatoma cells (Bel7402) with compounds **8**, **11** and **12** resulted in IC_50_ values between 4.8 and 7.4 nM [133]. The same group conducted a broader study in 2009, evaluating the cytotoxicity of compounds **8**–**13** against human malignant melanoma (B16-BL6), human sarcoma (HT-1080) and A2780 in addition to above mentioned cancer cells. The compounds **8**–**13** also showed 50% inhibition below 10 µg/mL. Furthermore, at concentration of 1 µg/mL, bromophenolics **10**, **11** and **12** induced a 77.5, 71.4 and 80.1% inhibition ratio of protein tyrosine kinase (PTK) with the overexpression of *c-kit*, which was identified in certain malignant diseases and presents a novel approach in cancer therapy [134].

Bis(2,3-dibromo-4,5-dihydroxybenzyl) ether **12** (BDDE), was isolated from the ethanolic extract of red alga, *Rhodomela confervoides*, alongside with three other bromophenols **15**–**17**. Structurally, all these compounds possess a single phenolic ring. Their cytotoxic activity was investigated against three human cancer cell lines, keratin-forming tumor cell line HeLa (KB), Bel7402 and A549, as well as normal, embryo lung fibroblasts (HELF). Compound **15** exhibited the strongest and most selective anticancer activity with IC_50_ values of 3.09, 3.18 and 3.54 µg/mL against KB, Bel7402 and A549 cells, respectively. Interestingly, bromophenol **17** induced stronger inhibition of proliferation against normal, HELF cells, while BDDE (**12**) was selectively active against KB cells only [135]. Liu and colleagues [136] synthesized bromophenol **12** and observed a potent and selective inhibitory activity against several types of cancer cells with determined IC_50_ values below 40 µg/mL. Compound **12** was the most active against human myelogenous leukemia cells (K562) since it inhibited 50% of proliferation at a concentration of 13.9 µg/mL [137]. The authors also performed a mechanistic study, which revealed that treatment with BDDE **12** modulated the level of pro- and anti-apoptotic proteins Bax and Bcl-2, respectively as well as caspases-3 and -9, indicating the induction of mitochondrial pathway-related apoptosis in K562 cells. The data obtained from the DNA relaxation assay, intercalation assay and molecular docking suggested that BDDE **12** acts as a topoisomerase I inhibitor by binding in the minor groove rather than intercalating into DNA [137]. However, since another study by Liu’s group [138] showed that **12** can intercalate in DNA, further evaluation is needed. The same group highlighted the potential use of compound **12** in cancer therapy, targeting angiogenesis since treatment with human endothelial cells (HUVEC) caused a decrease in the expression of VEGF and its receptor (VEGFR), associated with in vivo effects on zebrafish embryos, more specifically by inhibiting subintestinal vessel formation [139].

Marine algae, *L. nana* and *R. confervoides*, are natural sources of bis (2,3-dibromo-4,5-dihydroxy-phenyl)-methane (**14**) (BDDPM), which was extracted and synthesized by Wu and co-workers [140]. As reported by the authors, compound **14** exhibited a significant anti-proliferative activity against HeLa, RKO, HCT116, Bel7402 and U87 human cancer cell lines with IC_50_ values: 17.63, 11.37, 10.58, 8.7 and 23.69 µg/mL, respectively. Further studies using hepatoma cells (Bel7402) indicated that BDDPM **14** stimulated cell morphology change, mitochondrial-related apoptosis associated with the cleavages of caspases 3 and 9 and PARP, together with the inhibition of cell migration. The latter was concluded based on reduced wound healing and the inhibition of β1-integrin, known for mediating signal transduction between the cell and ECM. The authors suggested that **14** could be further developed into a novel anti-metastatic agent [140]. Similarly to BDDE **12**, BDDPM **14** inhibited angiogenesis in HUVEC cells which resulted from a broad inhibition of several receptor tyrosine kinases and reduced the production of NO which was proven to promote cancer cell invasiveness [141].

Other interesting bromophenol compounds are lanosol (**18**) and their derivatives, lanosol methyl ether (**19**), lanosol *n*-propyl ether (**21**) and lanosol aldehyde (**22**), which were isolated from red alga, *Polysiphonia lanosa* [142]. Lanosol ethyl ether (**20**) was later found to be an artefact resulting from fractionation over silica gel and using ethyl acetate, as concluded by Shoeib et al. [143]; however, here is presented the anticancer activity of **20** due to its contribution to the understanding of the structure–activity relationship. The authors screened bromophenols **18**–**22** against colon cancer cell lines, DLD-1 and HCT-116. Compounds **18**–**21** induced 50% of growth inhibition in DLD-1 cells at a concentration of 18.3, 14.6, 13.5 and 12.4 µM, respectively. On the other side, IC_50_ values determined after the treatment of HCT-116 cells with **18**–**21** were 20.4, 14.1, 2.51 and 1.32 µM, respectively. Therefore, it can be concluded that not only the presence of hydroxyl groups and bromine substituents affected the antitumor activity of the above-mentioned compounds, but also the length of the side chain. An increase in chain length led to a higher potency of the compounds, as can be observed for bromophenols **19** to **21**. Lanosol aldehyde **22** was the least active against DLD-1 (IC_50_ = 30.9 µM) and it was not tested against HCT-116 [143]. In 2009, a novel lanosol derivative, lanosol butanone (**23**), was isolated from another marine red alga, *Osmundaria colensoi*, and displayed significant anticancer activity against HL-60 cells (IC_50_ = 8.0 µM) [144].

Ma and colleagues extracted phenylethanol bromophenol (**24**), structurally similar to lanosol (**18**), and three unusual derivatives of **24**, bearing a sulfate group **25**–**27** also from the ethanolic extract of *R. confervoides* [145]. The authors reported moderate inhibitory activity of **24**–**27** against A549, A2780, BGC-823, Bel7402 and HCT-8 cancer cells, wherein the determined IC_50_ values were mostly in the range 12-21 µM. Notably, bromophenol sulfate (**25**) displayed the lowest IC_50_ value of 9.4 µM against human ovarian cancer [145].

In addition to the red and brown algae, the green species were also used for the isolation of interesting bromophenolic compounds. For example, the tropical green algae, *Avrainvillea nigricans*, was used by Colon and his team to isolate 3-bromo-4,5-dihydroxybenzyl alcohol (**28**). At a concentration of 8.9 µg/mL, **28** induced 50% inhibition of proliferation of KB cells, indicating moderate cytostatic activity. Structurally, the compound **28** differs from lanosol (**18**) in additional bromide substituent in *ortho* position to the benzylic alcohol [146]. Recently, extraction of another *Avrainvillea* species, *A. amadelpha*, resulted in the isolation of avrainvilleal (**29**) which exhibited moderate activity against HeLa cancer cells (IC_50_ = 9.64 ± 1.7 µM) [147]. On the other side, Wegener and Miller reported the first total synthesis of avrainvilleol, a similar compound to **29**, containing benzyl alcohol group [148].

### 3.3. Flavonoids

**Figure 3:**
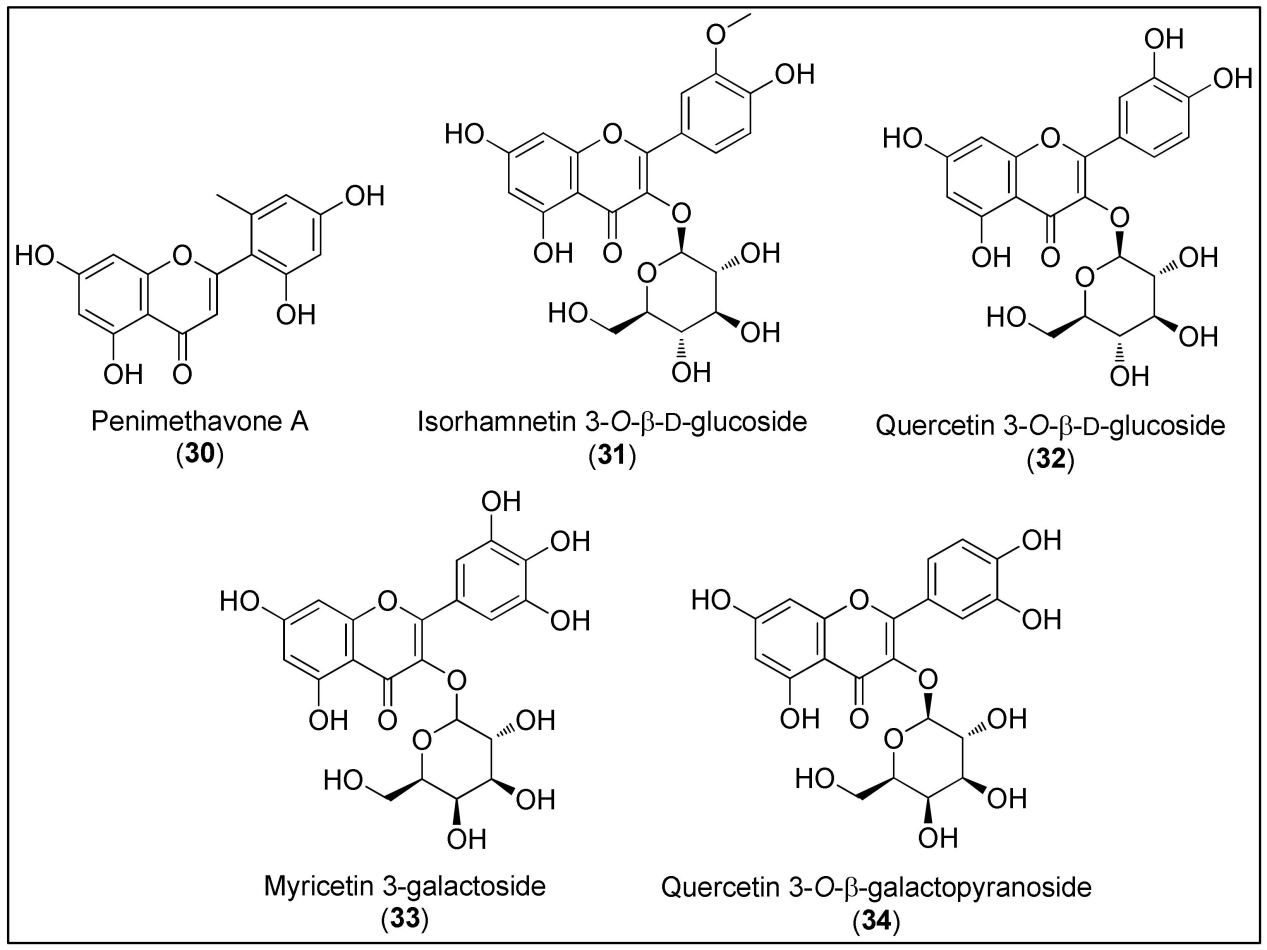
Chemical structures of flavonoids, penimethavone A (**30**), isolated from fungus *Penicillium chrysogenum* and flavonoid glycosides **31**–**34** isolated from the halophytes, *Salicornia herbacea* and *Limonium tetragonum*.

Flavonoids **30**–**34** (Figure 3) are characterized as a class of polyphenols with polyhydroxylated 2-phenylchromen-4-one backbone [149]. Except the simplest, penimethavone A (**30**), all the other members of this group **31**–**34** are monoglycosylated with glucose or galactose. Interestingly, marine-derived flavonoids are known to contain unusual substituents, including the methyl, chlorine, amino and sulfate groups. The latter are considered to be the result of ecological adaptation [150].

Compared to terrestrial flavonoids, marine congeners are noticeably less isolated, characterized and evaluated regarding biological activities [26,150,151]. As stated in an exhaustive review by Martins and co-workers [150], less than 100 flavonoids have been isolated, mostly from seagrasses and halophyte. However, molluscs, bacteria, fungi and corals were also reported as potential natural sources of these secondary metabolites. Since marine flavonoids have been acquainted to exhibit a broad spectrum of biological effects, such as antioxidant, antifouling, antitumor, antimicrobial and antidiabetic, these chemicals present a potential hit or lead compounds in the pharmaceutical industry. Martins et al. also emphasized that only a few dozen of flavonoids have been screened for biological activities [150].

Hou et al. [152] isolated a novel and unusual flavone, penimethavone A (**30**), from the fungus *Penicillium chrysogenum* derived from gorgonian coral *Carijoa* sp. The spectroscopic analysis revealed the presence of a methyl substituent at phenyl ring B, which is quite a rare group at this position among natural compounds. Shao’s group evaluated anticancer activity of **30** against human laryngeal epithelial cancer (Hep-2), A549, HeLa and rhabdomyosarcoma cells (RD). The last two cell lines were the most affected by penimethavone (**30**) with reported IC_50_ values of 8.41 and 8.18 µM, respectively [152].

Flavonoid glycosides, isorhamnetin-3-*O*-β-ᴅ-glucoside (**31**) and quercetin-3-*O*-β-ᴅ-glucoside (**32**), were isolated from a halophyte plant, *Salicornia herbacea**.*** Based on the results of Kong and co-workers, the authors suggested that compounds **31** and **32** can be used as chemopreventive agents [153]. The inhibitory activities of **31** and **32** on MMP-2 and MMP-9 in HT1080 cells, associated with an increase in TIMP-1 protein, which is an endogenous inhibitor of the aforementioned MMPs, were observed. A further mechanistic approach revealed the possible connection between the downregulation of MMP genes and suppressed AP-1 promoter activity [153]. Moreover, the cytostatic effect of **32** against HCT116 cells was reported (IC_50_ = 24.3 μg/mL) [154]. As stated by Mohammed et al., anticancer activity of flavonoid glycosides might be affected by the presence of sugar moiety and the number of hydroxyl groups, both increasing the hydrophilicity of metabolites and, additionally, making an internalization into cells difficult [154].

In 2017, a bioassay-guided fractionation of crude *Limonium tetragonum* extract resulted in the isolation of two known flavonoid glycosides, myricetin 3-galactoside (**33**) and quercetin 3-*O*-β-ᴅ-galactopyranoside (**34**). Both compounds **33** and **34** exerted inhibitory activities against MMPs in an HT1080 cell line in a selective manner. As observed for flavonoids **31** and **32**, the compounds **33** and **34** also suppressed the overexpression of MMP-2 and MMP-9 while simultaneously elevating TIMP-1 and TIMP-2 at the mRNA and protein levels. Compared to **33**, the mechanistic studies considering the MAPK signalling pathway revealed that compound **34** significantly reduces the levels of phosphorylated ERK and p-38 [155].

### 3.4. Coumarins

**Figure 4:**
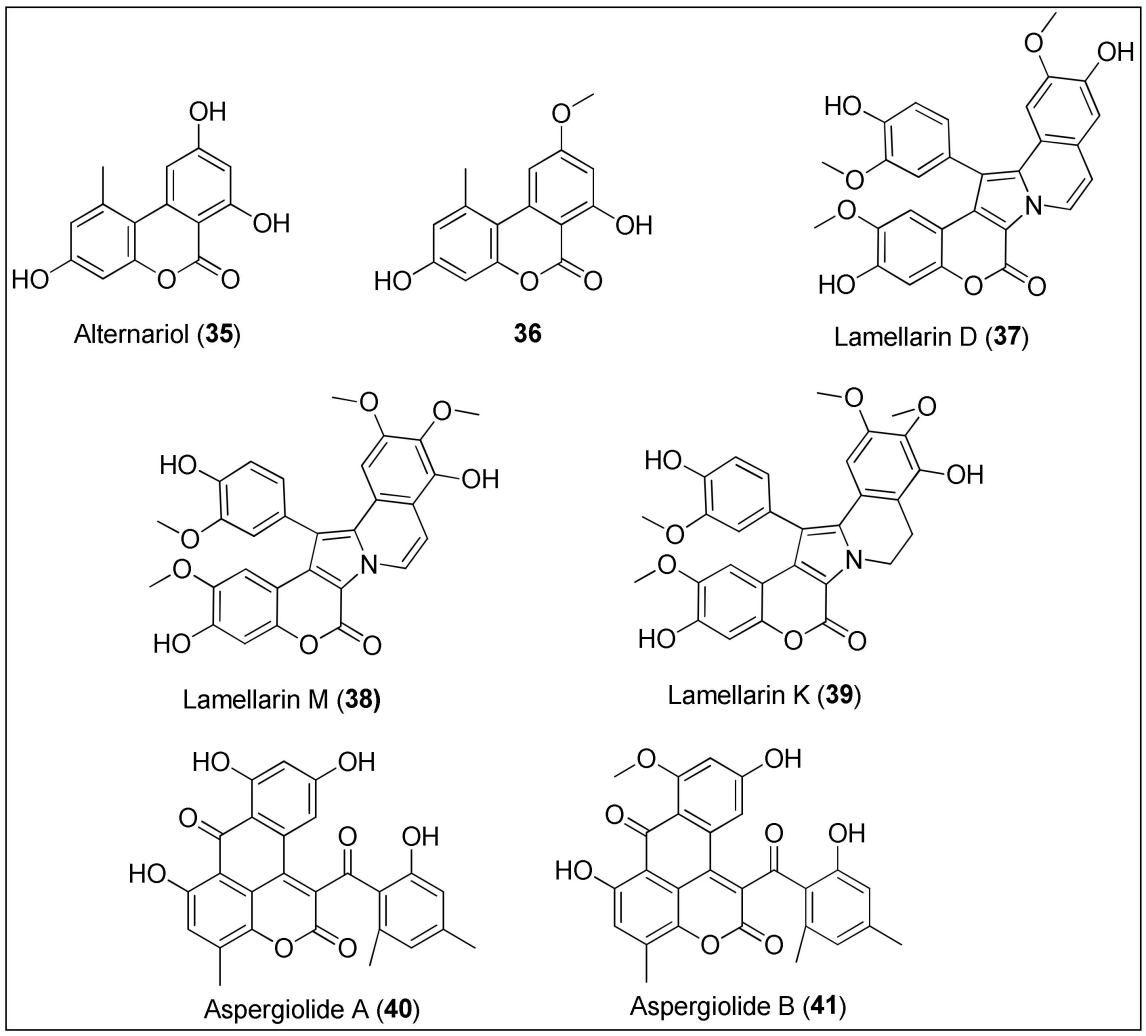
Chemical structures of coumarins: alternariol (**35**) and its analogue **36** isolated from the mangrove endophytic fungus 2240. Lamellarins D (**37**), M (**38**) and K (**39**) are obtained from the marine molluscs of the genus *Lamellaria*. Aspergiolides A (**40**) and B (**41**) are extracted from the marine fungus, *Aspergillus galucus*.

Coumarins **35**–**41** present a large and heterogenous class of naturally occurring phenolic compounds which can be found in higher plants, bacteria and fungi, as well as other marine organisms such as molluscs, tunicates or sponges (Figure 4) [156,157]. Structurally, they are characterized by a benzo-α-pyrone skeleton in which the aromatic is connected to a lactone ring [158]. Their role as secondary metabolites is to the protect from the herbivores, microorganisms and infection via antioxidative and enzyme inhibitory activities [156]. Numerous scientific studies have demonstrated miscellaneous pharmacological effects of coumarins, including antioxidative, cardioprotective, anti-inflammatory and anticancer activities [157,159]. The latter arises from their ability to inhibit the protein kinase and telomerase, induce a caspase-mediating apoptosis and exert an anti-angiogenic effect, as discussed by Önder [156]. Due to the simplicity of a benzopyrone skeleton accompanied with the possibility to easily obtain structural diversity by varying the degree of oxygenation, coumarins have attracted much attention from the medicinal chemists in order to develop new structural analogues for therapeutic utilization [160,161].

Tan et al. [162] isolated alternariol (**35**) and its derivatives from the mangrove endophytic fungus 2240 and evaluated their antiproliferative potential by means of an MTT assay against two epidermoid carcinoma cell lines, KB and KBv200, of which the latter is a multidrug resistant cell line. Besides alternariol (**35**), only the alternariol methyl ether (**36**) exhibited significant activity with IC_50_ values being 3.17, 3.12 and 4.82, 4.94 μg/mL for KB and KBv200, respectively. Interestingly, the authors observed a positive correlation between a number of hydroxyl groups and a strength of antitumor effect [162]. Both **35** and **36** were also isolated from the fungus, *Alternaria alterata*, residing in the soft coral, *Litophyton arboreum*. Alternariol (**35**) displayed anticancer properties against leukemia cell lines (L1210 and CCRF-CEM), while derivative **36** was active against colon and lung cancer cells (Colon-38 and H-125, respectively). However, both benzocoumarins **35** and **36** were toxic to the normal human cells, the granulocyte–macrophage progenitor (CFU-GM) [163].

Lamellarins are among the most interesting marine coumarins with a wide variety of biological activities. Lamellarins are characterized by a 14-phenyl-6*H*-[1] benzopyrano[40′,3′:4,5]pyrrolo[2,1-α] isoquinolin-6-one ring system and, because of the central pyrrole ring, can be also considered as alkaloids. Since 1985, more than 50 lamellarins have been discovered from various marine organisms. Lamellarins D (**37**), M (**38**) and K (**39**) are among the most cytotoxic, exhibiting IC_50_ values often in a nanomolar concentration range against different cancer cell lines [164,165]. For example, lamellarin D (**37**) was isolated from the marine molluscs of the genus *Lamellaria* and assayed against 12 human cancer cells, of which LNCaP, DU-145 and K562 were strongly affected, as demonstrated by the 50% growth inhibition concentrations in the 10–20 nM range. Furthermore, murine and human leukemia cells, both sensitive (P-388, CEM) and resistant (P388CPT5, CEM/C2) to camptothecin, respectively, were used for the evaluation of the correlation of topoisomerase I and cytotoxicity. At concentrations of 136 and 1482 nM, **37** inhibited growths of P-388 and P388CPT5 by 50%, respectively, while it was more active against human cell lines with determined IC_50_ values of 14 and 969 nM against CEM and CEM/C2, respectively. Lamellarin D (**37**) was found to be a novel topoisomerase I inhibitor due to intercalation into the DNA–topoisomerase I complex, resulting in its stabilization [166]. That was also observed by Ballot and her group [167] who reported another mode of antitumor activity by targeting cancer cell mitochondria [168,169]. As stated by the authors, compound **37** induced apoptosis of the above mentioned cell lines, P388 and topoisomerase I–mutated subclone P388CPT5 by increasing the levels of proapoptotic protein, Bax, and decreasing the expression of antiapoptotic proteins, Bcl-2 and cIAP2, along with caspase-3/-9 activation [167]. The great interest in this compound facilitated total syntheses of **37** and its structural analogues, together with the simultaneous evaluation of their biological activities [170,171]. Furthermore, lamellarins M (**38**) and K (**39**), found in the tunicates of the genus *Didemnum*, remarkably inhibited the growth of several cancer cell lines, more precisely, P388, multidrug-resistant P388 (Schabel), a wild type of chinese hamster ovary cells, CHO (AUXB1), AUXB1 cells resistant to doxorubicin (CCH^R^C5), A549, HT29, and human melanoma cells (MEL28). The measured 50% inhibitory concentrations were 0.15, 0.17, 0.07, 0.17, 0.06, 0.56, 0.54 and 0.19, 0.017, 0.19, 0.75, 0.18, 0.38, 0.40 μM for **38** and **39**, respectively [172]. Recently, some new lamellarins with specific structures and promising anticancer activities were discovered, as discussed by Vazquez-Rodriguez et al. [157]. 

In 2007, Du and co-workers first reported the isolation, structural characterization and biological evaluation of the anthraquinone derivative, aspergiolide A (**40**), derived from the marine fungus, *Aspergillus galucus*. At concentrations of 0.13, 0.28, 7.5 and 35 μM, **40** inhibited the growth of A-549, HL-60, BEL-7402 and P388 cells for 50%, respectively [173]. Considerably more detailed and extensive studies were conducted in 2014, investigating both the anticancer and pharmacokinetic properties of aspergiolide A (**40**). Compound **40** exhibited significant activity against 11 cancer cells with micromolar IC_50_ values (2.37–7.07 μM). The Western blot analysis showed that **40** induced caspase-mediated apoptosis of BEL-7402 cancer cells, related to the increase and decrease of Bax and Bcl-2 expressions, respectively. Further, the inhibition of DNA topoisomerase II by **40** was revealed, comparable to the positive control, adriamycin but with less toxic consequences. Finally, compound **40** successfully suppressed the growth of H22 and BEL-7402 cancer xenografts in mice without major effects on body weight [174]. A structural analogue of **40**, aspergiolide B (**41**), was also isolated by the group mentioned above from the same fungus species, *A. glaucus*. The authors observed that the *O*-methylation of the hydroxyl group at position C-8 did not affect the anticancer properties of **41** since they reported IC_50_ values of 0.24 and 0.51 μM against A-549 and HL-60 cancer cell lines, respectively [175]. Thus, the presence of the naphtho[1,2,3-*de*]chromene-2,7-dione structural feature, characteristic for both aspergiolides A (**40**) and B (**41**), might be used for the synthesis of new anticancer derivatives. Moreover, an in silico analysis indicated that **41** is a potential EGFR-TK inhibitor displaying low binding free energy in active site containing MET-766, THR-790 and THR-854 amino acid residues [176].

### 3.5. Terpenophenolics

**Figure 5:**
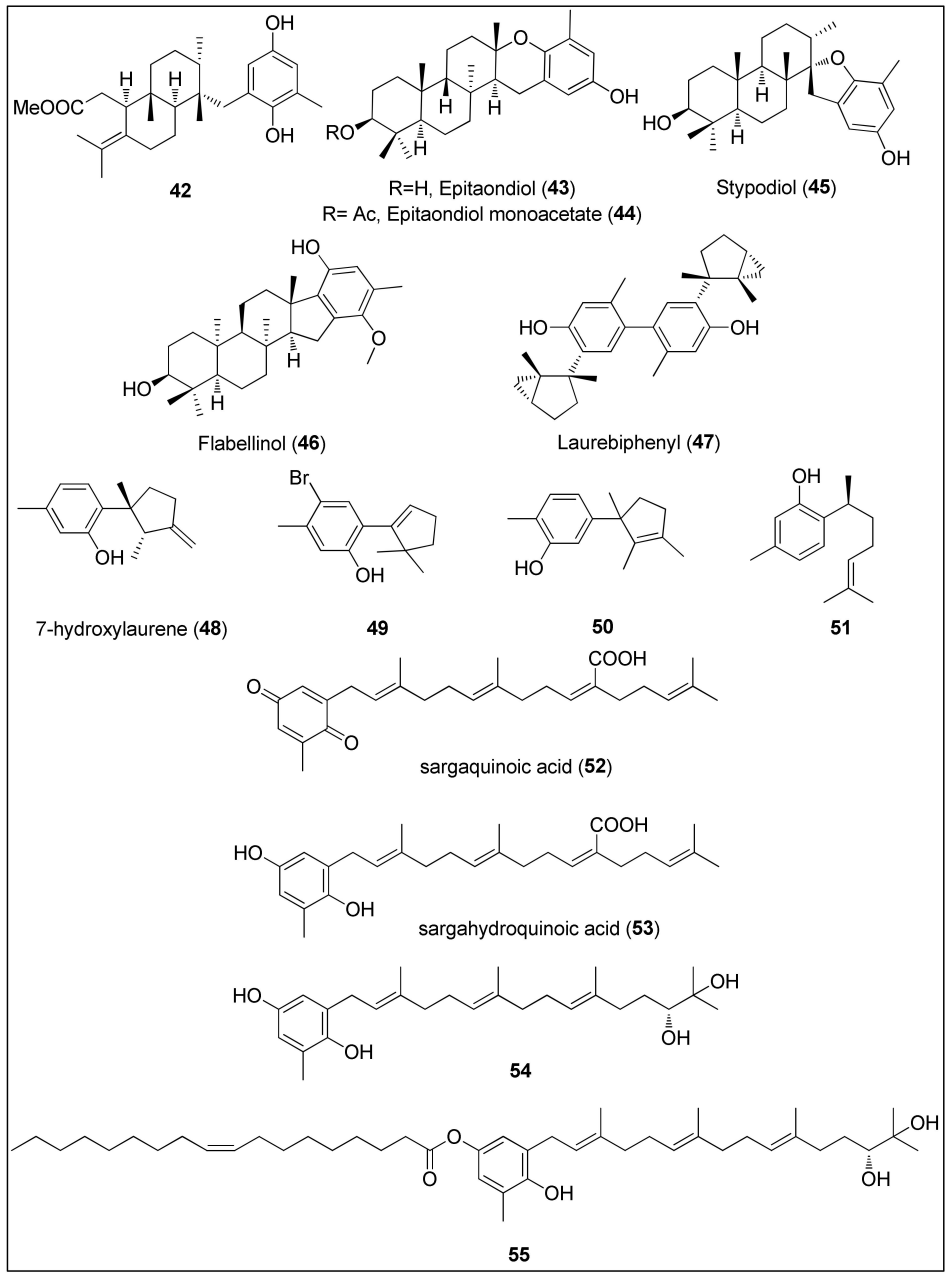
Chemical structures of meroditerpenes **42**–**47** isolated from brown algae (*Stypopodium* spp.). Sesquiterpenes **48**–**50** isolated from red algae (*Laurencia* spp.), **51** isolated from sponge, Didiscus flavus, and plastoquinones **52**–**55** isolated from brown algae *Sargassum* sp.

The structural complexity of marine secondary metabolites is manifested by the presence of terpenophenolic compounds, in particular, meroditerpenes **42**–**47** and merosesquiterpenes **48**–**51** (Figure 5). The first include chromenes, chromanols and plastoquinones which contain a hydroquinone skeleton linked to the side polyprenyl chain. The other hydroquinones will be discussed in the Section 3.6 [177]. The biosynthesis, structural features and biological activities of marine merosesquiterpenes were already extensively reviewed by Le Bideau et al. [178]. Their structures contain a phenolic core derived from the polyketide pathway and a unique, isoprenoid cyclic moiety. Although they can be isolated from marine sponges and gorgonian soft corals [179], brown macroalgae of the genus *Stypopodium* [180,181,182,183] and red seagrasses of the genus *Laurbncia* [184,185,186,187] are the main producers of these molecules. Like other marine-derived compounds, terpenophenolics differ in oxygenation and unsaturation levels as well as in the presence of one or more halogen atoms (Figure 5). Many studies have demonstrated their broad-spectrum of biological activities including ichthyotoxicity, insecticidal activity, tyrosine kinase inhibition, antimicrobial activity, microtubule inhibition and the antiproliferative effect against cancer cells [177,178,180,181,183].

Dorta and his colleagues used a brown alga, *Stypopodium zonale* from Macaronesia Archipelago, to extract terpenoids bearing phenolic moiety. Compound **42** displayed potent activity against HT-29, H-116 and A549 cells with IC_50_ values being 2.5 μg/mL or less [180]. Two other studies, using a different species of brown algae, *Stypopodium flabelliforme*, resulted in the isolation of the meroditerpenoids **43**–**46** [181,183]. Their anticancer activities were evaluated against several cancer and non-transformed cell lines by Pereira et al. [181]. The highest activity of almost 100% inhibition of cell proliferation was observed against human neuroblastoma cells (SH-SY5Y) for all compounds. Even though the authors did not provide the exact IC_50_ values, they can be clearly seen from the reported graphical representations as 6.25–12.5, <12.5 and 12.5–25 μM for epitaondiol (**43**), epitaondiol monoacetate (**44**) and stypodiol (**45**), respectively. Since the only difference between **43** and **44** is the absence and presence of the acetoxy group, it can be assumed that acetylation of **43** decreased biological activities. Interestingly, **43** and **44** were remarkably active against Chinese hamster fibroblasts (V79), unlike stypodiol (**45**), which showed little effect on normal cells [181]. The neurotoxicity of meroditerpenes from *S. flabelliforme* was also demonstrated by Sabry and co-workers [183]. They assayed flabellinol (**46**) against mouse neuroblastoma cell lines (Neuro-2a) which displayed LC_50_ values in the range from 2 to 11 μM. Moreover, at a concentration of 9 μM, **46** inhibited the growth by 50% of NCI-H460 cancer cells [183].

Shizuri and Yamada were first to isolate and determine the structure of dimeric sesquiterpene, laurebiphenyl (**47**), from the red algae, *Laurencia nidifica*, comprising of a unique, cyclolaurane-type of the skeleton [185]. Compound **47** was later extracted from *Laurencia tristricha*, collected from the Naozhou Island and tested against several cancer cell lines. Sun and co-workers [184] reported significant cytotoxicity of laurebiphenyl (**47**) against BGC-823, HeLa, A549, HCT-8 and Bel7402 cells with determined IC_50_ values of 1.22, 1.61, 1.68, 1.77 and 1.91 μg/mL, respectively. Roussis and his team used another *Laurencia* species, *L. microcladia* from the North Aegean Sea, to obtain novel cytotoxic sesquiterpenes **48**–**50**, which were published in two independent studies [186,187]. 7-Hydroxylaurene (**48**) exhibited an antiproliferative effect against human cancer cell lines (MCF-7, PC3, A431, HeLa and K562) as well as CHO, inducing 50% of proliferation inhibition at concentrations of 15.8, 18.1, 23.9, 40.5, 64.2 and 78.2 μM, respectively. As hypothesized by the authors, the presence of an exocyclic-methylene group might be responsible for the observed biological activity [186]. Finally, sesquiterpenes **49** and sterochemically undefined **50** exhibited mild antiproliferative activities against lung cancer cells, NSCLC-N6 (IC_50_ = 73.4 and 83.7 μM) and A549 (IC_50_ = 52.4 and 81.0 μM), respectively. The authors assumed that the presence of the hydroxyl group and the unsaturated cyclopentenyl moiety led to higher activity while the bromine did not remarkably affect the overall results [187].

Another bisabolane-type sesquiterpene phenol, (+)-curcuphenol (**51**), was firstly isolated from the marine sponge, *Didiscus flavus*, in 1987 by Wright et al. [179]. The compound **51** was also found in another marine sponge, *Myrmekioderma styx*, as well as a terrestrial plant, *Baccharis genistelloides* [188]. At a concentration of 7 μg/mL, **51** induced 50% inhibition of P-388 cells, while the minimum inhibitory concentrations against A-549, MDA-MB and HCT-8 were determined as 10, 0.1 and 0.1 μg/mL, respectively [188].

Further, **51** demonstrated moderate antiproliferative activity against four HCT-116 cells (with or without the expression of the p53 and p21 genes). Its activity did not depend on the p53 nor p21 mechanism since IC_50_ values of 27, 33, 33 and 35 μg/mL were determined for p53^+/+^, p53^−/−^, p21^+/+^ and p21^−/−^, respectively [189]. Finally, Rodrigo and her colleagues used CaCo-2 colon cancer cells to evaluate the action mechanism of (+)-curcuphenol (**51**). They showed that this secondary metabolite inhibited the proliferation and DNA synthesis associated with the induction of apoptosis via caspase-3 activation [188].

The organic extract of an Australian marine brown alga, *Sargassum fallax*, was used to isolate plastoquinones, sargaquinoic acid (**52**) and sargahydroquinoic acid (**53**). Both **52** and **53** exhibited prominent antitumor activity with IC_50_ values of 17 and 14 μM against P388 cells, respectively [190]. A previous study of Hur and colleagues had revealed that sargaquinoic acid (**52**) induced caspase-mediated apoptosis in a human keratinocyte cell line, HaCaT, while it had no effect on the Bcl-2 and Bax proteins’ expressions [191]. On the other side, plastoquinones **54** and **55** obtained from another *Sargassum* species, *S. micracanthum*, displayed significantly higher and comparable antiproliferative activity against the murine colon 26-L5 adenocarcinoma cell line (IC_50_ = 1.51 and 1.69 μg/mL, respectively) [192].

### 3.6. Quinones and Hydroquinones

**Figure 6:**
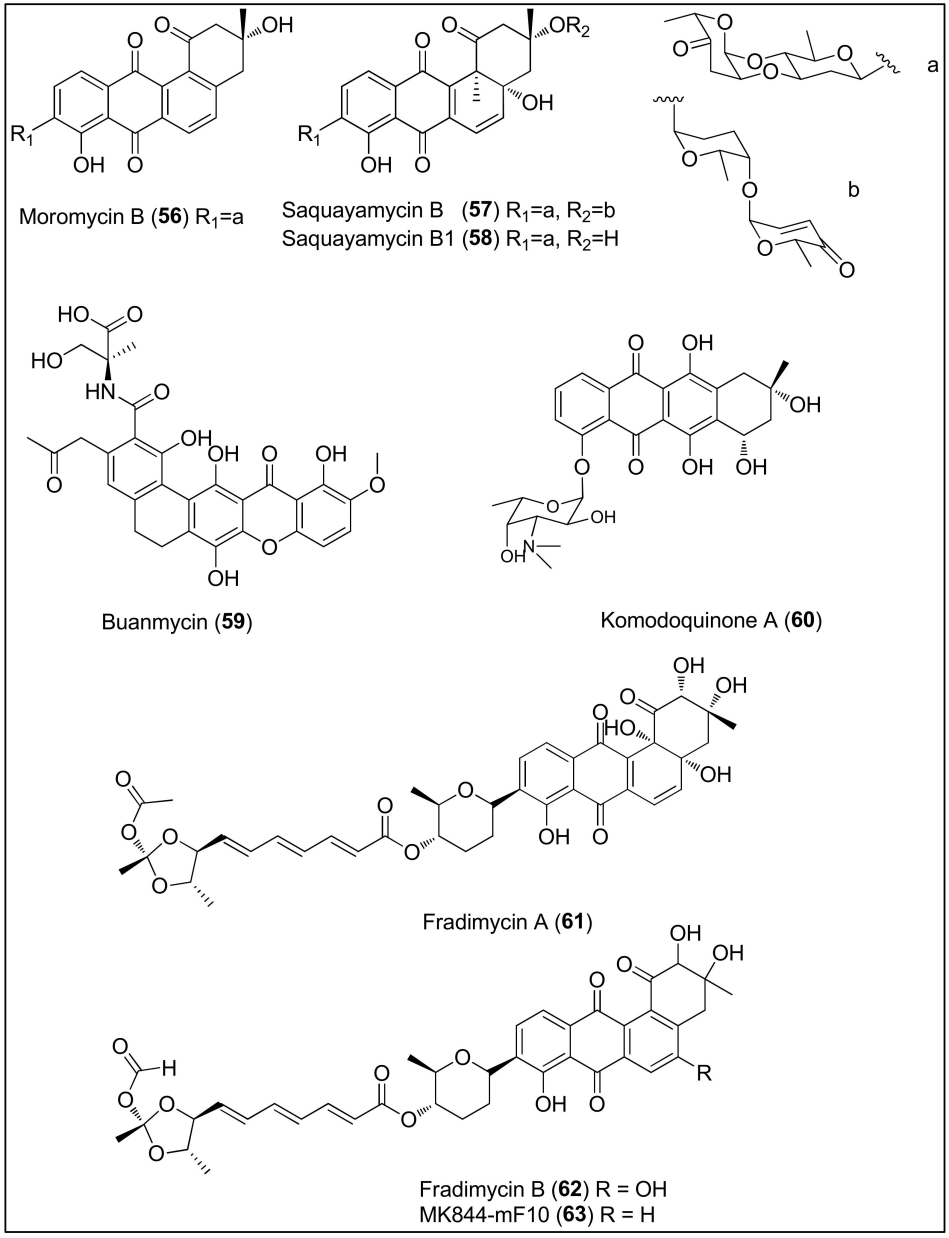
Chemical structures of moromycin B (**56**), saquayamycins B (**57**) and B*1* (**58**) isolated from the *Streptomyces* sp. OC1610.4 strain, buanmycin (**59**), isolated from the *Streptomyces* strain, SNR69, komodoquinone A (**60**), isolated from the *Streptomyces* sp. KS3 strain and fradimycin A (**61**), fradimycin B (**62**) and MK844-mF10 (**63**), isolated from the *Streptomyces fradiae* strain PTZ0025.

**Figure 7:**
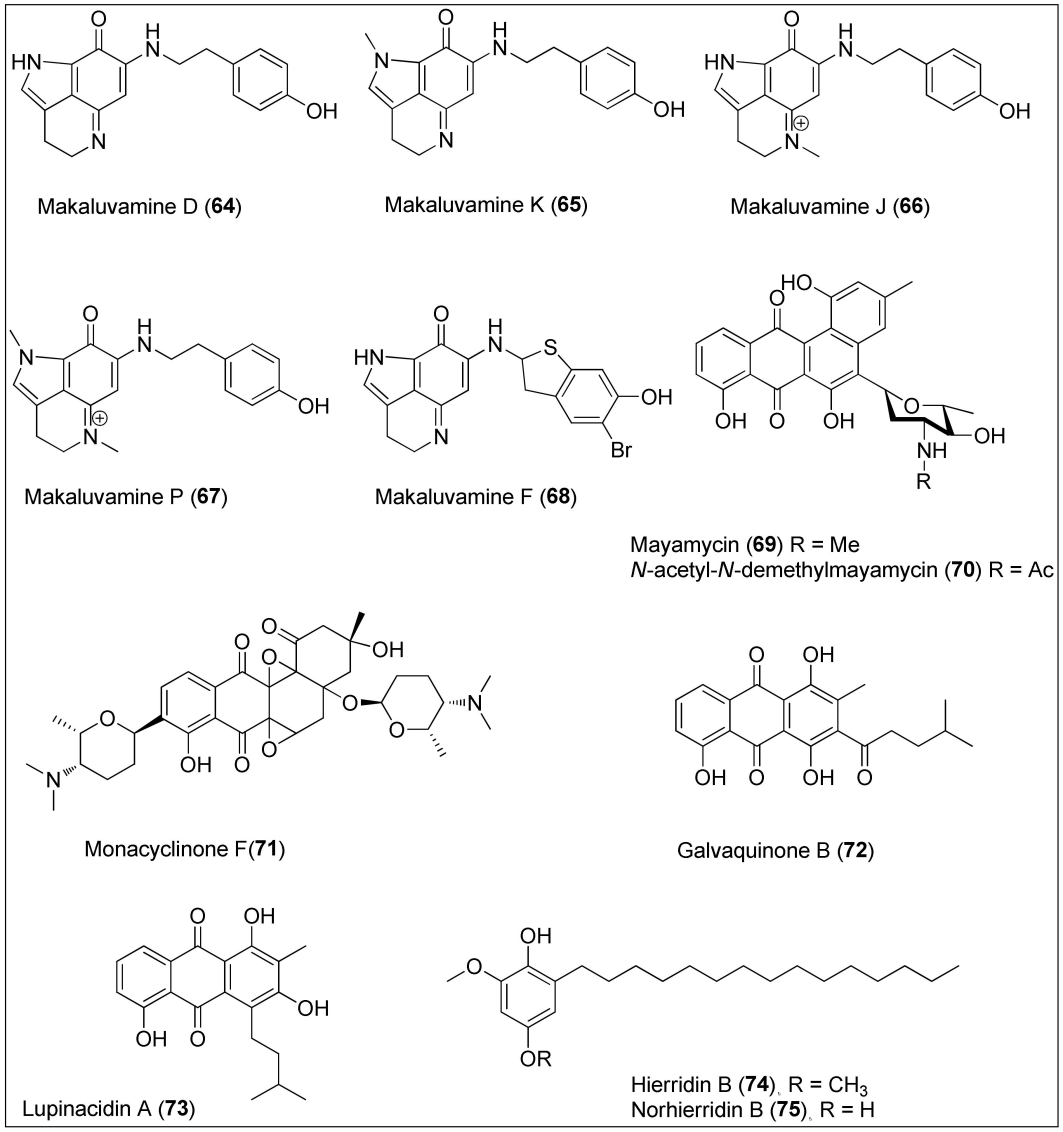
Makaluvamines D (**64**), K (**65**), J (**66**), P (**67**) and F (**68**) isolated from the two sponge genera of *Zyzzya* and *Latrunculi,* mayamycin (**69**) and N-acetyl-N-demethylmayamycin (**70**) isolated from the *Streptomyces* sp. strains HB202 and 182SMLY, respectively; monacyclinone F (**71**) isolated from the *Streptomyces* sp. strain M7_15, galvaquinone B (**72**) and lupinacidin A (**73**) isolated from the *Streptomyces spinoverrucosus* and hierridin B (**74**) isolated from the *Phormidium ectocarpi* and *Cyanobium* sp. and its structural analogue, norhierrdin B (**75**).

Quinones possess a conjugated cyclic dione function and hydroquinones are their reduced derivatives. They belong to the aromatic organic compounds and can be obtained by the oxidation processes of certain phenolic molecules. They differ in carbon skeletons and might be formed by inter- and intramolecular cyclizations further linked to specific amino acids residues or carbohydrate units. In addition, quinone and hydroquinone moieties can also be present in terpenes and terpenoids, of which some were discussed in the previous section (vide supra) that resulted in a rather challenging classification [193,194]. Chemical structures of quinones and hidroquinones originating from marine species, mainly *Streptomyces* sp., are presented in Figure 6 and Figure 7.

The broth medium of the marine-derived *Streptomyces* sp. OC1610.4 strain was used to extract angucycline glycosides, moromycin B (**56**), saquayamycins B (**57**) and B1 (**58**), consisting of tetrangomycin core *C*- or *O*-linked to one or two deoxysugar units (Figure 6). Cytotoxicity assays revealed that all three compounds, **56**–**58**, remarkably reduced the proliferation of breast cancer cells (MCF-7, MDA-MB-231 and BT-474) in a sub-micromolar range (0.16–0.67 μM), comparable to the standard control, doxorubicin. Furthermore, transwell and wound-healing assays showed potential antimetastatic properties of **57** since it reduced the invasion and migration of MDA-MB-231 cells at concentrations of 25 and 50 nM [195].

Another marine *Streptomyces* strain, SNR69, which was later found to be the most similar to *Streptomyces cyaneus*, was collected from a tidal mud in Buan (Republic of Korea) and identified as a producer of novel, pentacyclic buanmycin (**59**) that exerted both antibacterial and cytotoxic properties. As reported by the authors, **59** strongly inhibited 50% of the proliferation of A549, HCT116, SNU638, SK-HEP1 and MDA-MB-231 cells at concentrations of 1.7, 0.9, 0.8, 1.9 and 1.2 μM, respectively, similarly to etoposide used as a positive control. Contrary, **59** showed no activity towards the K562 cell line [196].

Itoh and co-workers isolated and determined the absolute stereochemistry of komodoquinone A (**60**) from the marine *Streptomyces* sp. KS3 strain. It is an anthracycline containing an amino sugar connected to the ring system and bearing a unique methyl substituent at position 9. As shown, compound **60** can induce morphological changes and lead to neuritogenic activity against the neuroblastoma cell line (Neuro 2A). Interestingly, the cell cycle of Neuro 2A cells was arrested at the G1 phase, contrary to other anthracycline antibiotics which intercalate in DNA, indicating a different mechanism of action of **60**. The authors assumed that the carbohydrate moiety might be a prerequisite for the observed biological results since the aglycon part was weakly active [197,198].

In 2012, a study performed by Xin and colleagues using the marine *Streptomyces fradiae* strain, PTZ0025, led to the isolation of the capoamycin-type antibiotics **61**, **62** and **63,** characterized by a benz[a]anthraquinone core linked to a deoxysugar and polyenyl side chain [199]. Moreover, another *Streptomyces fradiae* strain, BDMS1, was also found to produce the abovementioned metabolites [200]. As reported by the authors, fradimycin A (**61**), fradimycin B (**62**) and analogue MK844-mF10 (**63**) exhibited potent inhibitory activity in vitro, against human colon cancer cells (HCT-15 and SW620) and rat glioma cells (C6). Compound **62** was the most active, displaying IC_50_ values of 0.13, 4.33 and 0.47 μM against HCT-15, SW620 and C6, respectively. For that reason, the mechanism of action of compound **62** was further studied and it was found that **62** induced cell cycle arrest at the G0/G1 phase associated with an increase of apoptotic and necrotic cells [199].

Many studies were performed on interesting pyrroloiminoquinone alkaloids containing a makaluvamine-type scaffold and phenolic substituent, which were isolated from the two sponge genera of *Zyzzya* and *Latrunculia* collected in the Pacific–Oceania region. In addition, a significant cytotoxicity of makaluvamines has been reported through interaction with topoisomerase II resulting in DNA cleavage [201,202]. Among them, makaluvamines D (**64**), K (**65**), J (**66**) and P (**67**) were found to be the most active against the PANC-1 cell line, exhibiting IC_50_ values of 0.29, 0.56, 0.054 and 0.3 μM, respectively. Structure–activity relationship studies revealed three main structural properties responsible for potent anticancer activity: a conjugation system in the main makaluvamine core, the presence of a cationic tetrahydropyridinium moiety, and a tyramineyl substituent. Furthermore, since makaluvamine J (**66**) also had an IC_50_ value in a nanomolar range (120 nM) against the ovarian cancer cell line OVCAR-5, the authors decided to continue with preclinical studies by using **66** [203]. Despite the existence of a planar structure and a positive charge that contributes to the high affinity for DNA, already in 2005, Dijoux and her colleagues showed that intercalation into DNA is not the only mode of its action [204]. On the other side, structurally different and stereochemically undefined makaluvamine F (**68**) also displayed strong inhibitory activity in a sub-micromolar range against HCT-116 with a determined IC_50_ value of 0.17 μM [205]. Further, in 2016, Goey et al. found that this natural product reduced the activity of HIF-1α and its downstream target, VEGF indicating the possible role of makaluvamines in hypoxia conditions [206]. Synthetic approaches in the preparation of some makaluvamines, as well as their structural analogues, were also reported [207,208,209].

The culture broth of the *Streptomyces* sp. strain HB202, derived from the marine sponge, *Halichondria panicea*, was used to isolate and characterize mayamycin (**69**), a novel benz[a]anthracene derivative. More precisely, **69** is known for its unique *C*-bounded angolosamine unit, with a dimethylamino group at the C-5 of the skeleton. Mayamycin (**69**) exhibited potent activity with determined IC_50_ values of 0.2, 0.3, 0.2, 0.16, 0.29, 0.13, 0.15 and 0.33 μM against eight cancer cell lines, HepG2, HT-29, GXF251L, LXF529L, MAXF401NL, MEXF462NL, PAXF1657L and RXF486L, respectively. As published by the authors, **69** was also cytotoxic toward a mouse fibroblast cell line (NIH-3T3) [210]. An analogue of **69**, *N*-acetyl-*N*-demethylmayamycin (**70**), was obtained in 2016 from another marine *Streptomyces* strain, 182SMLY, by Liang et al. [211]. The authors reported that treatment with **70** resulted in 50% of proliferation inhibition at the concentrations of 0.7, 1.4, 3.9 and 0.5 μM, against U251, U87-MG, SHG-44 and C6 glioma cell lines, respectively, as determined by sulforhodamine B assay. Furthermore, it was shown that **70** can induce apoptosis in U251 cells [211]. Synthetic routes toward mayamycin have been discussed and published emphasizing intramolecular aldol condensation and Hauser annulation as key steps [212,213].

In 2015, six new angucyclinone derivatives were extracted from the sponge-derived *Streptomyces* sp. Strain, M7_15, of which monacyclinone F (**71**) showed the highest activity against rhabdomycosarcoma cancer cells (SJCRH30) displaying an EC_50_ value of 0.73 μM. The authors concluded that the structural characteristic arising from the presence of two epoxide rings, aminodeoxysugar and ketone moiety could be of great importance for biological activity [214].

Anthraquinones with alkyl substituents, galvaquinone B (**72**) and lupinacidin A (**73**), were isolated from the marine-derived *Streptomyces spinoverrucosus* by Hu and colleagues [215]. They screened both metabolites for cytotoxic activity against Calu-3 and H2887 cancer cell lines and reported IC_50_ values of 5.0 and 12.2 μM for **72** and 8.8 and 3.1 μM for **73**, respectively. Recently, Sottorff and his team isolated both **72** and **73** from the sea anemone (*Gyractis sesere*) from Easter Island, but further confirmed the Actinobacteria of the genus *Verrucosispora* as the exact producer of those compounds [216].

In 1998, Papendorf and his colleagues used the marine cyanobacterium *Phormidium ectocarpi* to isolate hierrdin B (**74**), a methylated hydrouinone with a long aliphatic chain which showed antiplasmodial activity [217]. However, its cytotoxic potential was examined against a panel of human cancer cell lines only in 2013. Leão et al. purified **74** from the marine picocyanobacterium *Cyanobium* sp. LEGE 06113, and determined its selective, but weak, activity towards HT-29 cells with an IC_50_ value of 100.2 µM [218]. The same group used the aforementioned cells to evaluate the mechanism underlying the biological activity of hierrdin B (**74**). The authors pointed out that **74** targets mitochondrial activity by increasing the mRNA expression of VDAC1, a key protein involved in mitochondria-mediated apoptosis. That observation was accompanied with the inhibition of cell cycle progression induced by **74** [219]. In the meantime, an analogue of the natural product **74**, norhierridin B (**75**), was synthesized with improved inhibitory activity against several cancer cell lines by activating the p53 pathway. The IC_50_ values of 0.61, 0.77, 0.68, 2.0, 0.61 and 3.2 µM against MDA-MB-231, SKBR3, MDA-MB-468, A375, Huh-7 and HCT116 were measured, respectively. Therefore, the authors suggested that the presence of two hydroxy groups in the quinone skeleton is of great importance for the improvement of anticancer activity [220].

### 3.7. Miscellaneous Compounds

**Figure 8:**
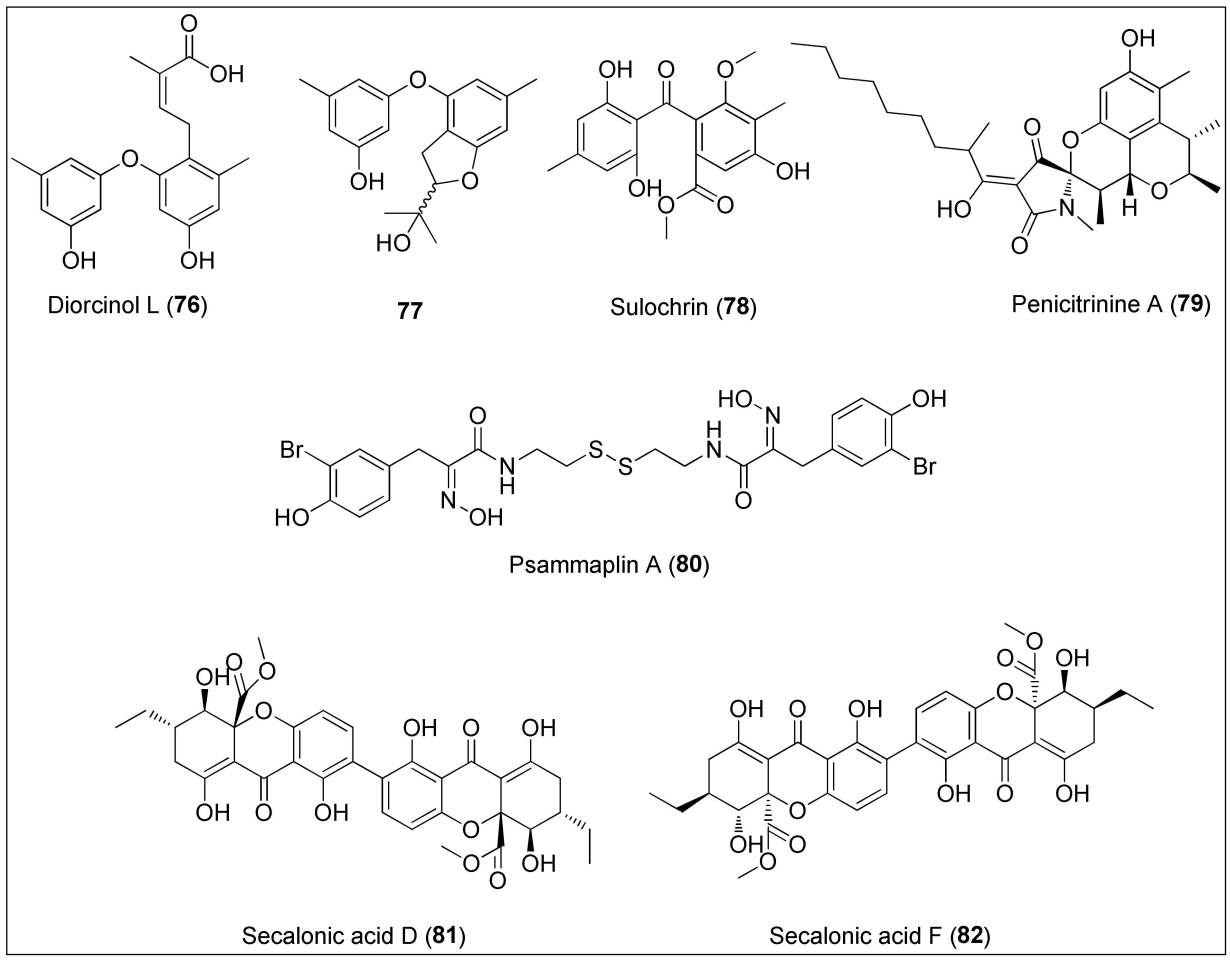
Diorcinol L (**76**) isolated from the fungus of the genus *Didymellaceae* and its derivative **77**, sulochrin (**78**) obtained from the fungus *Aspergillus falconensis*, penicitrinine A (**79**) isolated from the *Penicillium citrinum*, psammaplin A (**80**) extracted from the marine sponge of the genus *Pseudoceratina*, and secalonic acids D (**81**) and F (**82**) obtained from *Penicillium* sp. and *Aspergillus aculeatus*.

The ethyl-acetate extract of the sponge-derived fungus of the genus *Didymellaceae* was used to isolate several phenol derivatives including diorcinol L (**76**) (Figure 8). It exhibited potent antiproliferative activity against Huh-7, DU145, HeLa and HL60 cancer cell lines with determined IC_50_ values of 5.7, 9.1, 7.1 and 9.6 µM, respectively [221]. Interestingly, strong activity against cancer cells including DU145 and HeLa of **76**, isolated from the endophytic algae-derived fungus, *Aspergillus tennesseensis*, was not observed by Zhang et al. However, the authors reported that the dihydrobenzofuran derivative **77** displayed activity towards THP-1 cells since it inhibited the 50% growth of the cells at the concentration of 7.0 µg/mL [222]. On the other side, the benzophenone derivative, sulochrin (**78**), a compound somewhat similar to diorcinol L (**76**), was very recently isolated from the Red Sea-derived fungus, *Aspergillus falconensis* [223]. At a concentration of 5.1 µM, **78** inhibited 50% growth of a mouse lymphoma cell line (L5178Y), while MDA-MB-231 cell migration was inhibited at 70 µM. The authors also performed docking studies which revealed inhibition activity of **78** against the CDK-2, TOP-2 and MMP-13 proteins.

Penicitrinine A (**79**) is a phenolic derivative with a unique spiro skeleton that was isolated from the marine fungus *Penicillium citrinum*. It showed a promising and moderate activity against various solid tumor types in vitro, with A-375 cells being the most sensitive. At the concentrations of 30.88, 12.78 and 7.06 µM, a treatment with **79** for 24 h, 48 h and 72 h, respectively, resulted in 50% growth inhibition of the abovementioned cell line. Furthermore, Liu and co-workers investigated the mechanism of action in detail regarding apoptotic and metastatic activity. The authors revealed that penicitrinine A (**79**) induced apoptosis of A-375 cells by decreasing and increasing the expression of the Bcl-2 and Bax proteins, respectively. Additionally, **79** induced cell migration suppression by downregulating MMP-9 and upregulating TIMP-1 levels [224].

An unusual bromotyrosine metabolite containing oxime and disulfide moieties, psammaplin A (**80**), was isolated from the marine sponge of the genus *Pseudoceratina* [225]. Several studies have demonstrated anticancer effects of this disulfide dimer, exerted both in vitro and in vivo, as recently extensively reviewed by Jing and co-workers [225]. In brief, the cytotoxicity of **80** is manifested in the regulation of the expression of proteins involved in angiogenesis, DNA replication, apoptosis, proliferation and invasion. Therefore, it has been revealed that psammaplin A (**80**) inhibits aminopeptidase N (APN), mycothiol-S-conjugate amidase (MCA), topoisomerase II, farnesyl protein transferase, histone deacetylases (HDACs) and leucine aminopeptidase [225]. More importantly, synthetic approaches were developed to prepare both **80** and its analogues, which were found as more potent inhibitors of HDACs [226] and DOT1L (disruptor of telomeric silencing-1 like) than the natural product **80** [227].

Tang et al. used the culture extract of the marine-derived *Penicillium oxalicum* to extract secalonic acid D (**81**). The authors demonstrated selective cytotoxic effects of **81** towards PANC-1 cells (IC_50_ value of 0.6 µM) which were adapted to certain nutrient conditions associated with the assumption of the mechanism of action via the inhibition of the Akt signalling pathway [228]. Furthermore, **81** displayed potent cytotoxicity against both sensitive and multidrug resistant cells with determined IC_50_ values being: 6.8, 6.4, 5.3, 4.9, 5.1 and 4.9 µM against S1, S1-MI-80, H460, H460/MX20, MCF-7 and MCF-7/ADR, respectively. In addition, **81** was found to induce cell death through c-Jun/Src/STAT3 signalling by inhibiting the proteasome-dependent degradation of c-Jun [229]. Another research, performed by Guru and colleagues, used secalonic acid D (**81**), however, isolated from the terrestrial source. It was revealed that **81** exhibited antitumor activity in both normal (HUVEC) and MCF-7 cancer cells through the Akt/mTOR/p70S6K pathway resulting in the inhibition of eNOS and ERK phosphorylation together with MMP degradation as key pro-angiogenesis factors [230].

On the other hand, an isomer of **81**, secalonic acid F (**82**), has been isolated from the marine-derived fungal strains, *Penicillium* sp. F11 and *Aspergillus aculeatus* [231,232]. A study by Li and colleagues [232] revealed 50% of growth inhibition of HL-60 cells at the concentration of 4.1 µg/mL, further associated with induction of apoptosis via caspase-3 activation and the modulation of the RhoGDI2 protein. The latter is connected to the invasion and metastasis, thus being recognized as a novel therapeutic approach in cancer treatment [233]. Further, a phenolic derivative **82** was more potent towards HepG2 cells than the positive control (5-FU), exhibiting IC_50_ values of: 45.5, 8.7 and 7.7 µM after 24, 48 and 72 h treatment, respectively. Those observations were, in relation to mitochondrial-mediated apoptosis, more precisely the activation of caspases-3 and -9. The authors also performed in vivo studies that demonstrated lower tumor weights after treatment with **82** [231]**.** A new biological evaluation of secalonic acid F (**82**) resulted in the identification of a potential target, MARCH1, for the treatment of hepatocellular carcinoma, in which the downregulation suppressed the migration and invasion of HepG2 and Hep3B cancer cells [234]. Finally, the most recent study obtained by Özenver and co-workers demonstrated the potency and selective toxicity of **82** against leukemia and multiple myeloma cells with regard to normal cells mediated through apoptosis and necrosis, as well as tubulin disassembly [235].

## 4. Conclusions

Due to novel strategies and methods of the sampling of organisms from harsh marine environments and the constant progress in technologies and methodologies of extraction, purification, identification and characterization of natural products, the complex and diverse marine flora and fauna have been more and more accessible and recognized as a rich source of secondary metabolites with often unique chemical structures specific only for the marine environment. Although sometimes confusing as the same compound can be categorized to more than one class, marine natural products have been traditionally classified as peptides, alkaloids, macrocylclic polyethers, terpenoids and steroids and phenolics. Especially the latter have been widely studied in terrestrial organisms, mainly plants, while systematic investigations of marine phenolic compounds connecting their isolations, chemical characterizations and biological evaluations are still very scarce. The bioproduction of phenolics in marine species is triggered by both biotic and abiotic stress, with the purpose to quickly respond and to adapt to the varying ecosystem conditions. For that reason, the difference in the phenolic content could differ depending on geographical distribution, time and season of collection, as well as on the applied laboratory methodology of extraction and purification. Furthermore, despite the known main biosynthetic pathways for the biosynthesis of phenolic compounds, i.e., shikimic acid, the malonate-acetate and mevalonate-acetate, the complete biosynthesis of phenolic compounds is often specific to one or several marine species and it may imply postbiosynthetic modifications by symbiotic organisms.

Natural products of a marine origin have displayed a broad range of pharmaceutically important biological effects. Especially, marine-derived phenolic metabolites and their derivatives or analogues have attracted great attention due to antioxidant, anti-inflammatory, antimicrobial and anticancer activities resulting from their abilities to inhibit reactive radicals through the single electron and hydrogen atom transfer reactions, or to interact and to bond to target proteins and other biologically important molecules.

In this review are summarized anticancer potentials of recently isolated highly active marine phenolic compounds as determined through in vitro, in vivo or in silico assays. The most promising cytotoxic marine-derived phenolics and their biologial activites are depicted in Table 1. As shown, marine phenolic compounds inhibited cancer cells proliferation, in some cases at sub-micromolar or nanomolar concentrations (lamellarins D (**37**), M (**38**), K (**39**), aspergiolide B (**41**), fradimycin B (**62**), makulavamine J (**66**), mayamycin (**69**), *N*-acetyl-*N*-demethylmayamycin (**70**) or norhierridin B (**75**)). Furthermore, they exhibited anticancer properties by the induction of apoptosis or the inhibition of cell migration and invasive potential. Finally, phlorotannins **1**–**7** and bromophenols **12**–**29** represent groups of the most studied phenolic compounds, of which the former are recognized as protective agents against UVB or gamma radiation-induced skin damages.

In order to obtain clinical evidence about the anticancer effects of marine phenolic compounds, these promising results should be further evaluated in in vivo studies in cancer patients. It is also of great significance to develop new bioprospecting programs and to promote further technological advancements in sampling from harsh marine environments. The improvements in the isolation and characterization of natural products present in traces and novel chemical synthetic strategies to overcome the scarcity of phenolic secondary metabolites from the marine matrices as the main limiting factor have to be also achieved. Taking this into consideration, it is expected that research in the field of marine phenolic compounds will become increasingly important and will more and more contribute to the future arsenal of anticancer agents.

## Figures and Tables

**Table 1:** Anticancer properties of marine-derived phenolic compounds obtained by different experimental systems (in vitro, in vivo or in silico).

Compound	Class	Anticancer Properties Displayed by In Vitro, In Vivo or In Silico Experiments	Ref.
Phloroglucinol **1**	Phlorotannin	Inhibition of migration and capillary-tube formation resulting in reduction of Lewis lung carcinoma in mouse model	[79]
		50% of MDA-MB-231 cells’ migration at 50 µM by inhibiting epithelial-mesenchymal cell transition via downregulation of SLUG protein	[80]
		Apoptosis of the HT-29 cells through insulin-like growth factor 1 receptor (IGF-1R) and inhibition of downstream proteins	[81,82]
		Upregulation of pro-apoptotic proteins Bax and Bad and downregulation of anti-apoptotic Bcl-2 and Bcl-xL	[81]
		Activity against breast cancer stem-like cells by reducing the expression of CD44, Oct4, Notch2, β-catenin and Sox2 proteins, associated with enhanced sensitivity of cells to the ionizing radiation and standard chemotherapeutic drugs (taxol, cisplatin and etoposide)	[83]
		The higher sensitization of colon cancer cells, HT-29 and HCT116, to 5-fluorouracil (5-FU)	[84]
Eckol **2**		Exhibition of radioprotective properties in vitro by ROS scavenging, and in vivo through the reduction of side effects caused by gamma ray-irradiation	[85,86]
		Decreased expression of the stem-like glioma cells markers, CD133, Nestin and Musashi-1	[87]
		Increased sensitivity of stem-like glioma cells to cytotoxic prodrug, temozolomide as well as to ionizing radiation via PI3K⁄Akt and Ras⁄Raf-1⁄ERK signalling pathways	
		Inhibition of the pancreatic cancer cell line (SW1990) growth	[88]
		Increase of cytotoxic T-lymphocytes required for annihilation of sarcoma (S180) xenograft in mouse model	[90]
Phlorofucofuroeckol **3**		Decreased HCT116, HT-29, LoVo and SW480 cells’ viabilities for 38%, 31%, 47% and 90%, respectively, at 100 μM	[92]
		Induction of apoptosis and enhanced expression of activating transcription factor 3 (ATF3)	
		Suppression of migration and invasion of MCF-7 and MDA-MB-231 cells by downregulation of Nf-κB and Toll-like receptor 4 (TLR-4) alongside with reducing MMP-2 and -9 expression	[93]
Dieckol **4**		Suppression of migration and invasion of MCF-7 and MDA-MB-231 cells by downregulation of Nf-κB and Toll-like receptor 4 (TLR-4) alongside with reducing MMP-2 and -9 expression	[93]
		Regulation of human fibrosarcoma cells’ (HT1080) invasion via downregulation of MMP-2 and -9 expression through Nf-κB signalling	[95]
		Inhibition of SK-Hep1 cells’ migration through downregulation of MMP-9 expression	[96]
		Decrease of B16F0 melanoma cells via downregulation of Ras-related C3 botulinum toxin substrate 1 (Rac1) and Wiskott–Aldrich syndrome protein family member 2 (WAVE2)	[98]
		Suppression of HT1080 cells migration via reduced expression and phosphorylation of focal adhesion kinase (FAK)	[99]
		Induction of apoptosis in Hep3B hepatocellular carinoma cell line through cytochrome *c* release and caspase cascade	[101]
		Binding to MMP-2, MMP-9, MAPK, ERK and p38 via hydrogen bonds revealed by computational studies	[102]
		Suppression of ovarian cancer cells (A2780, SKOV3) growth by inducing caspase-dependent and regulating AKT and p38 signaling	[103]
		Reduced volume of SKOV3 tumor xenograft in mouse model	
		Reducing mobility of MCF-7 cells by increasing MMP inhibitors, TIMP-1 and -2, as well as degrading extracellular matrix	[104]
		Increase in Bax/Bcl-2 ratio indicating mitochondrial-mediated apoptosis in SK-BR-3 (breast cancer) and PANC-1 (pancreatic cancer) cells	[105,109]
		Protective and chemopreventing effects against hepatocellular carcinoma (HCC) in rats via upregulation of VEGF, MMP-2/9, PCNA and COX-2	[106,107]
		Anti-migratory and apoptotic activity in A549 cells associated with inhibition of Pi3K/AKT/mTOR signalling pathways and activation of tumor-suppressor, E-cadherin	[108]
		Protection from gamma radiation and consequent damage, both in vitro and in vivo	[110,111]
Diphlorethohydroxycarmalol **5**		Activity against murine leukemia (P-388) and vincristine-resistant cells (IC_50_ = 10.5 and 8.0 µg/mL, respectively)	[112]
		Induction of apoptosis in HL60 cells via upregulation of Bax, downregulation of Bcl-2 and increased expression of cleaved caspase-3 and cleaved PARP	[113]
		Photoprotective activity against UVB radiation and side effects, through absorption of radiation, inhibition of MMPs’ expression and scavenging of ROS	[115,116,117,118]
Dioxinodehydroeckol **6**		Induction of apoptosis in MCF-7 cells via downregulation of p65, IKK (IκB kinase) and NIK (NF-κB inducing kinase) Upregulation of Bax and downregulation of Bcl-2	[119]
		Downregulation of tyrosinase and tyrosinase-linked proteins (TRP-1 and TRP-2) as well as MITF (microphthalmia-associated transcription factor), involved in regulation of melanin formation in B16F10 mealnoma cells	[121]
		Protection against radiation-induced skin damage	[122]
Fucodiphloroethol G **7**		Activity against HeLa, A549, HT1080 and HT29 cancer cells (IC_50_ = 298.2, 226.5, 242.5 and 228.5 µM, respectively)	[124]
		Suppression of MAPK and Akt signalling pathway necessary for expression of proteinases (MMP-2, MMP-9 and APN) involved in degradation of extracellular matrix	[126]
Bis (2,3-dibromo-4,5-dihydroxybenzyl) ether (BDDE) **12**	Bromophenol	Actvity against human myelogenous leukemia cells (K562) (IC_50_ = 13.9 µg/mL)	[137]
		Induction of mitochondrial pathway-related apoptosis via modulation of Bax, Bcl-2, caspases-3 and -9 in K562 cells	
		Topoisomerase I inhibitor by binding in the minor groove of DNA	
		Repression of angiogenesis through decrease in the VEGF and VEGFR expression in HUVEC cells associated with inhibition of subintestinal vessel formation in vivo on zebrafish embryos	[139]
Bis (2,3-dibromo-4,5-dihydroxy-phenyl)-methane (BDDPM) **14**		Anti-proliferative activity against HeLa, RKO, HCT116, Bel7402 and U87 human cancer cell lines (IC_50_ = 17.63, 11.37, 10.58, 8.7 and 23.69 µg/mL, respectively)	[140]
		Mitochondrial-related apoptosis associated with the cleavages of caspases 3 and 9 and PARP	
		Inhibition of β1-integrin leading to suppression of Bel7402 cell migration and invasion	
		Inhibition of angiogenesis in HUVEC cells through inhibition of several receptor tyrosine kinases and reducing cell invasiveness by lowering the production of NO	[141]
**18**–**21**		Antiproliferative activity against DLD-1 (IC_50_ = 18.3, 14.6, 13.5 and 12.4 µM) and HCT-116 cells (IC_50_ = 20.4, 14.1, 2.51 and 1.32 µM)	[143]
Lanosol butanone **23**		Antiproliferative activity against HL-60 cells (IC_50_ = 8.0 µM)	[144]
Bromophenol sulfate **25**		Antiproliferative activity against A2780 cancer cells (IC_50_ = 9.4 µM)	[145]
Avrainvilleal **29**		Moderate activity against HeLa cancer cells (IC_50_ = 9.64 ± 1.7 µM)	[147]
Penimethavone A **30**	Flavonoid	Antiproliferative activity against HeLa and RD cancer cells (IC_50_ = 8.41 and 8.18 µM)	[152]
Isorhamnetin-3-*O*-β-ᴅ-glucoside **31**		Inhibition of MMP-2 and MMP-9 in Ht1080 cells, associated with increase in TIMP-1 protein	[153]
Quercetin-3-*O*-β-ᴅ-glucoside **32**			
		Antiproliferative activity against HCT116 cancer cells (IC_50_ = 24.3 µM)	[154]
Myricetin 3-galactoside **33**		Suppression of MMP-2 and MMP-9 expression while simultaneously elevating TIMP-1 and TIMP-2 at the mRNA and protein levels in HT1080 cell line	[155]
Quercetin 3-*O*-β-ᴅ-galactopyranoside **34**			
		Reduced the levels of phosphorylated ERK and p-38 in HT1080 cell lines	
Alternariol **35**	Coumarins	Antiproliferative activity against KB and KBv200 cancer cells (IC_50_ = 3.17 and 4.82 µM)	[162]
		Anticancer properties against leukemia cell lines (L1210 and CCRF-CEM)	[163]
Alternariol methyl ether **36**		Antiproliferative activity against KB and KBv200 cancer cells (IC_50_ = 3.12 and 4.94 µM)	[162]
		Antiproliferative properties against leukemia cell lines (Colon-38 and H-125)	[163]
Lamellarin D **37**		Antiproliferative activity against LNCaP, DU-145 and K562 cancer cells in the nanomolar range	[164,165]
		Antiproliferative activity against CEM and CEM/C2 lines (14 and 969 nM)	[166]
		Topoisomerase I inhibitor due to intercalation at the DNA–topoisomerase I complex	
		Induction of apoptosis of P388 and P388CPT5 (topoisomerase I—mutated subclone) cells via increasing the levels of Bax and decreasing the expression of Bcl-2 and cIAP2 along with caspase-3/-9 activation	[167]
Lamellarin M **38**		Antiproliferative activity against P388, Schabel, AUXB1, CCH^R^C5, A549, HT29 and MEL28 cancer cells (IC_50_ = 0.15, 0.17, 0.07, 0.17, 0.06, 0.56 and 0.54 µM)	[172]
Lamellarin K **39**		Antiproliferative activity against P388, Schabel, AUXB1, CCH^R^C5, A549, HT29 and MEL28 cancer cells (IC_50_ = 0.19, 0.017, 0.19, 0.75, 0.18, 0.38 and 0.40 µM)	
Aspergiolide A **40**		Antiproliferative activity against A-549, HL-60, BEL-7402 and P388 cancer cells (IC_50_ = 0.13, 0.28, 7.5 and 35 µM)	[173]
		Activity against 11 cancer cells with micromolar IC_50_ values (2.37–7.07 μM)	[174]
		Induction of caspase-mediated apoptosis of BEL-7402 cancer cells via modulation of Bax and Bcl-2 expression	
		Inhibition of DNA topoisomerase II	
		Suppressing the increase of H22 and BEL-7402 cancer xenografts in mice without major effects on body weight	
Aspergiolide B **41**		Antiproliferative activity against A-549 and HL-60 cancer cells (IC_50_ = 0.24 and 0.51 µM)	[175]
		Potential EGFR-TK inhibitor displaying low binding free energy in active site containing MET-766, THR-790 and THR-854 amino acid residues	[176]
Epitaondiol (**43**), epitaondiol monoacetate (**44**) and stypodiol (**45**)	Terpenophenolics	The highest activity of almost 100% inhibition of cell proliferation was observed against human neuroblastoma cells (SH-SY5Y)	[181]
Flabellinol **46**		Antiproliferative activity against mouse neuroblastoma cell line (Neuro-2a) and NCI-H460 cells (IC_50_ = 9 µM)	[183]
Laurebiphenyl **47**		Antiproliferative activity against BGC-823, HeLa, A549, HCT-8 and Bel7402 cancer cells (IC_50_ = 1.22, 1.61, 1.68, 1.77 and 1.91 µM)	[184]
7-Hydroxylaurene **48**		Antiproliferative activity against MCF-7, PC3, A431, HeLa and K562, as well as CHO cancer cells (IC_50_ = 15.8, 18.1, 23.9, 40.5, 64.2 and 78.2 µM)	[186]
(+)-curcuphenol **51**		Antiproliferative activity against P388 cancer cells (IC_50_ = 7 µg/mL)	[178]
		Antiproliferative activity against p53^+/+^, p53^−/−^, p21^+/+^ and p21^−/−^ cancer cells (IC_50_ = 27, 33, 33 and 35 µg/mL)	[189]
		Inhibition of CaCo-2 colon proliferation and DNA synthesis associated with induction of apoptosis via caspase-3 activation	[188]
Sargaquinoic acid (**52**), sargahydroquinoic acid (**53**)		Antiproliferative activity against P388 cancer cells (IC_50_ = 17 and 14 µM)	[190]
Plastoquinones **54** and **55**		Antiproliferative activity against murine colon 26-L5 adenocarcinoma cell line (IC_50_ = 1.51 and 1.69 μg/mL)	[192]
Moromycin B (**56**), saquayamycins B (**57**) and B1 (**58**)	Quinones and hydroquinones	Inhibition of proliferation of breast cancer cells (MCF-7, MDA-MB-231 and BT-474) in sub-micromolar range (0.16–0.67 μM)	[195]
Saquayamycin B (**57**)		Suppression of MDA-MB-231 cells invasion and migration at concentrations of 25 and 50 nM for 12 h	
Buanmycin **59**		Antiproliferative activity against A549, HCT116, SNU638, SK-HEP1 and MDA-MB-231 cancer cells (IC_50_ = 1.7, 0.9, 0.8, 1.9 and 1.2 μM)	[196]
Komodoquinone A **60**		Morphological changes and neuritogenic activity against neuroblastoma cell line (Neuro 2A)	[197,198]
		Induction of Neuro 2A cell cycle arrest at G1 phase	
Fradimycin B **62**		Antiproliferative activity against HCT-15, SW620 and C6 cancer cells (IC_50_ = 0.13, 4.33 and 0.47 μM)	[199]
		Induction of cell cycle arrest at G0/G1 phase associated with an increase of apoptotic and necrotic cells	
Makaluvamine J **66**		Antiproliferative activity against PANC-1 (IC_50_ = 0.054 μM) and OVCAR-5 cancer cells (IC_50_ = 120 nM)	[203]
Makaluvamine F **68**		Antiproliferative activity against HCT-116 (IC_50_ = 0.17 μM)	[205]
		Role in hypoxia conditions via inhibition of HIF-1α and its downstream target, VEGF	[206]
Mayamycin **69**		Antiproliferative activity against eight cancer cell lines: HepG2, HT-29, GXF251L, LXF529L, MAXF401NL, MEXF462NL, PAXF1657L and RXF486L (IC_50_ = 0.2, 0.3, 0.2, 0.16, 0.29, 0.13, 0.15 and 0.33 μM)	[210]
*N*-acetyl-*N*-demethylmayamycin **70**		Antiproliferative activity against U251, U87-MG, SHG-44 and C6 glioma cell lines (IC_50_ = 0.7, 1.4, 3.9 and 0.5 μM)	[211]
		Induction of apoptosis in U251 cells	
Monacyclinone F **71**		Antiproliferative activity against rhabdomycosarcoma cancer cells (SJCRH30) (EC_50_ = 0.73 μM)	[214]
Galvaquinone B **72**		Antiproliferative activity against Calu-3 and H2887 cancer cells (SJCRH30) (IC_50_ = 5.0 and 12.2 μM)	[216]
Lupinacidin A **73**		Antiproliferative activity against Calu-3 and H2887 cancer cells (SJCRH30) (IC_50_ = 8.8 and 3.1 μM)	
Hierrdin B **74**		Antiproliferative activity against HT-29 cancer cells (IC_50_ = 100 μM)	[218]
		Targeting mitochondrial activity by increasing mRNA expression of VDAC1 accompanied with inhibition of cell cycle progression	[219]
Norhierridin B **75**		Antiproliferative activity against MDA-MB-231, SKBR3, MDA-MB-468, A375, Huh-7 and HCT116 cancer cells (IC_50_ = 0.61, 0.77, 0.68, 2.0, 0.61 and 3.2 μM)	[220]
Diorcinol L **76**	Miscellaneous	Antiproliferative activity against Huh-7, DU145, HeLa and HL60 cancer cells (IC_50_ = 5.7, 9.1, 7.1 and 9.6 µM)	[221]
Sulochrin **78**		Antiproliferative activity against L5178Y cancer cells (IC_50_ = 5.1 µM)	[223]
		Inhibition of MDA-MB-231 cell migration	
		Inhibition of CDK-2, TOP-2 and MMP-13 proteins	
Penicitrinine A **79**		Antiproliferative activity against A-375 cancer cells (IC_50_ = 30.88, 12.78 and 7.06 µM, after treatment for 24 h, 48 h and 72 h, respectively)	[224]
		Induction of apoptosis of A-375 cells by modulation of Bcl-2 and Bax expression	
		Suppression of A-375 cell migration by downregulating MMP-9 and upregulating TIMP-1 levels	
Psammaplin A **80**		Inhibition of aminopeptidase N (APN), mycothiol-S-conjugate amidase (MCA), topoisomerase II, farnesyl protein transferase, histone deacetylase (HDAC) and leucine aminopeptidase, involved in angiogenesis, DNA replication, apoptosis, proliferation and invasion	[225]
Secalonic acid D **81**		Antiproliferative activity against PANC-1, S1, S1-MI-80, H460, H460/MX20, MCF-7 and MCF-7/ADR cancer cells (IC_50_ = 0.6, 6.8, 6.4, 5.3, 4.9, 5.1 and 4.9 µM)	[228,229]
		Induction of cell death through c-Jun/Src/STAT3 signalling by upregulation of c-Jun expression	[229]
		Inhibition of eNOS and ERK phosphorylation together with MMP degradation via Akt/mTOR/p70S6K pathway	[230]
Secalonic acid F **82**		Antiproliferative activity against HL-60 cancer cells (IC_50_ = 4.1 µg/mL)	[232]
		Induction of apoptosis via caspase-3 activation and modulation of RhoGDI2 protein	
		Antiproliferative activity against HepG2 cancer cells (IC_50_ = 45.4, 8.7 and 7.7 µM, after treatment for 24 h, 48 h and 72 h, respectively)	
		Cell cycle arrest at the G1 phase and apoptosis induction activating caspases-3 and -9	
		Suppression of HepG2 and Hep3B cells invasion via downregulation of MARCH1	[234]
		Toxicity towards leukemia and multiple myeloma cells mediated through apoptosis and necrosis as well as tubulin disassembly	[235]

## Data Availability

Not applicable.

## List of Abbreviations

26-L5	murine colon adenocarcinoma cell line
3T3-L1	mouse embryonic fibroblast cell line
4CL	4-coumaroyl CoA ligase
A2780	human ovarian cancer cell line
A375	human melanoma cell line
A431	epidermoid carcinoma cell line
A549	adenocarcinomic human alveolar basal epithelial cell line
A5490	airway epithelial cells and mitochondrial DNA depleted cancer cell line
AKT	serine/threonine-specific protein kinase (PKB)
AP-1	transcription factor
APN	aminopeptidase N
ATF3	Activating Transcription Factor 3
AUXB1	wild type of chinese hamster ovary cells, CHO
B16-BL6	murine melanoma cell line
B16F0	murine melanoma cell line, parent cells
B16F10	murine melanoma cell line from a C57BL/6J mouse
Bad	Bcl2-associated agonist of cell death
Bax	Bcl-2-associated X protein
Bcl-2	B-cell lymphoma 2
Bcl-xL	B-cell lymphoma-extra large
BDDE	bis (2,3-dibromo-4,5-dihydroxybenzyl) ether
BDDPM	bis (2,3-dibromo-4,5-dihydroxy-phenyl)-methane
Bel7402	human hepatocellular carcinoma cell line
BGC-823	human gastric carcinoma cell line
BT-474	human breast carcinoma are characterized by the overexpression of human epidermal growth factor receptors 2 (HER-2) and estrogen receptors (ER)
C4H	cinnamate 4-hydroxlyase
C6	rat glioma cell line
Caco2	human colorectal adenocarcinoma cells
Calu-3	human lung cancer cell line
CCH^R^C5	AUXB1 cells resistant to doxorubicin
CCRF-CEM	leukemia cell line
CD44	cell-surface glycoprotein
CDK2	Cyclin-dependent Kinase 2
CEM	line of lymphoblastic cells originally derived from a child with acute lymphoblastic leukemia
CEM/C2	camptothecin (CPT) resistant derivative of the human T cell leukemia cell line CCRF-CEM
CFU-GM	granulocyte–macrophage progenitor (GMP)
cIAP2	baculoviral IAP repeat-containing protein 3
c-Jun	proto-oncogene, transcription factor AP-1
Colon-38	human colon cancer cell line
COX-2	cyclooxigenase-2
DLD-1	human colon cancer cell line
DOT1L	disruptor of telomeric silencing-1 like
DU-145	human prostate cancer cell line
EA.hy926	human umbilical vein endothelial cells
ECM	Extracellular Matrix
ECV-304	human umbilical vein endothelial cells
EGFR-TK	Epidermal Growth Factor Receptor Tyrosine Kinase
eNOS	Nitric Oxide Synthase, endothelial
ERK	Extracellular signal-Regulated Kinases
GXF251L	human gastric carcinoma cells
H-125	human lung cancer cell line
H22	murine hepatic carcinoma cell line
H2887	non-small-cell lung cancer cell line
H460	large cell lung cancer cell line
H460/MX20	large cell lung cancer cell line, derived from H460, mitoxantrone induced ABCG2-overexpressing cells
HaCaT	human keratinocyte cells
HCC	hepatocellular carcinoma
HCT116	human colon cancer cell line
HCT-15	human colon adenocarcinoma cell line
HCT-8	human ileocaecal adenocarcinoma cell line
HDAC	histone deacetylase
HeLa	human cervical cancer cell line
HELF	embryo lung fibroblasts
Hep3B	human hepatoma cell line
HepG2	human hepatoma cell line
HIF-1α	hypoxia-inducible factor 1-alpha
HL-60	leukemia cells
HT-1080	fibrosarcoma cell line
HT-29	human colon cancer cell line
Huh-7	human hepatoma cell lines
HUVEC	human endothelial cells
IFN-γ	Interferon gamma
IGF-1R	Insulin-like Growth Factor 1 Receptor
IKK	IκB kinase
K562	human myelogenous leukemia cells
KB	epidermoid carcinoma cell lines
KBv200	epidermoid carcinoma cell lines, multi-drug resistant (MDR) cell line
L1210	leukemia cells
L5178Y	human leukemia monocytic cell line
LNCaP	androgen-sensitive human prostate adenocarcinoma cell line
LoVo	human colon cancer cell line, supraclavicular lymph node metastasis
LXF529L	lung cancer cell line
MAPK	mitogen-activated protein kinase
MARCHF1	E3 ubiquitin-protein ligase MARCHF1
MAXF401NL	human mammary cancer cell line
MCA	Mycothiol-S-conjugate Amidase
MCF-7	human breast cancer cell line
MCF-7/ADR	Adriamycin-resistant human breast cancer cell line
MDA-MB-231	epithelial, human breast cancer cell line
MEL28	human melanoma cell line
MEXF462NL	human melanoma cell line
MITF	microphthalmia-associated transcription factor
MMP3	Matrix Metalloproteinase-3
MRC-5	normal fibroblast cell line
mTOR	Mammalian Target of Rapamycin
MTT	3-(4,5-dimethylthiazol-2-yl)-2,5-diphenyl tetrazolium bromide, reagent
NCI-H460	human lung carcinoma epithelial cells
NDEA	N-nitrosodiethylamine
Neuro-2a	neuroblastoma cell line
NF-κB	Nuclear Factor kappa-light-chain-enhancer of activated B cells
NIH-3T3	murine fibroblast cell line
NIK	NF-κB inducing kinase
Notch2	Neurogenic Locus Notch Homolog Protein 2
NSCLC-N6	non-small cell lung cancer cell line
Oct4	Octamer-binding transcription factor 4
OVCAR-5	human epithelial ovary carcinoma cell line
P-338	sensitive murine leukemia cells
p38	mitogen-activated protein kinases
P-388	bipotential murine pre-B cell lymphoma
P388CPT5	murine leukemia cells resistant to the reference topoisomerase I poison camptothecin (CPT)
p70S6K	ribosomal protein S6 kinase beta-1
PAL	Phenylalanine Ammonia-Lyase
PANC-1	human pancreatic cancer cell line
PARP	Poly (ADP-ribose) Polymerase
PAXF1657L	human pancreatic tumor cell line
PC3	bone metastasis of a grade IV prostatic adenocarcinoma cell line
PCNA	proliferating cell nuclear antigen
PI3K	Phosphoinositide 3-Kinase
PKS	Polyketide Synthase
Rac1	Ras-related C3 botulinum toxin substrate 1
RAF	Rapidly Accelerated Fibrosarcoma, protein kinase
RAS	Rat Sarcoma Virus, GTP-ase
RD	human rhabdomyosarcoma cell line
Reg3A	regenerating family member 3 alpha
Reg3g	regenerating islet-derived protein 3 gamma
RhoGDI2	Rho GDP dissociation inhibitor 2
RKO	colon carcinoma cell line
ROS	Reactive Oxygen Species
RXF486L	human renal cancer cell line
S1	human colon cancer cell line
S1-M1-80	human colon cancer cell line, derived from S1 cells, mitoxantrone-selected ABCG2-overexpressing cells
SHG-44	human glioma cell line
SH-SY5Y	cell line human neuroblast from neural tissue
SJCRH30	rhabdomycosarcoma cancer cell line
SKBR3	human breast cancer cell line, hypertriploid
SK-Hep1	human hepatic adenocarcinoma cell line
SKOV3	ovarian carcinoma cells
SLUG	transcription factor (SNAI2)
SNU638	human stomach carcinoma cell line
Sox2	(sex determining region Y)-box 2, trancription factor
Src	proto-oncogene tyrosine-protein kinase Src
STAT3	Signal Transducer and Activator of Transcription 3
SW1990	spleen metastasis of a grade II pancreatic adenocarcinoma
SW480	primary colon adenocarcinoma cell line
TAL	Tyrosine Ammonia Lyase
THP-1	human leukemia monocytic cell line
TIMP-1	TIMP Metallopeptidase Inhibitor 1
TLR-4	Toll-like receptor 4
TNF-α	Tumor Necrosis Factor alpha
TOP-2	DNA topoisomerase 2-alpha
TRP-1	tyrosinase and tyrosinase-linked proteins
TRP-2	tyrosinase and tyrosinase-linked proteins
U251	glioblastoma-derived human cell line
U87	human primary glioblastoma cell line, synonym: U87-MG
V79	chinese hamster fibroblasts
VDAC1	Voltage-dependent Anion-selective Channel 1
VEGF	Vascular Endothelial Growth Factor
VEGFR	Vascular Endothelial Growth Factor Receptor
WAVE2	Wiskott–Aldrich syndrome protein family member 2
WST-1	cell proliferation reagent
